# High level accumulation of EPA and DHA in field‐grown transgenic Camelina – a multi‐territory evaluation of TAG accumulation and heterogeneity

**DOI:** 10.1111/pbi.13385

**Published:** 2020-05-08

**Authors:** Lihua Han, Sarah Usher, Sjur Sandgrind, Kirsty Hassall, Olga Sayanova, Louise V. Michaelson, Richard P. Haslam, Johnathan A. Napier

**Affiliations:** ^1^ Department of Plant Sciences Rothamsted Research Harpenden Herts UK; ^2^ Present address: Department of Plant Breeding Swedish University of Agricultural Sciences Alnarp Sweden

**Keywords:** omega‐3, Camelina, metabolic engineering, GM field trials

## Abstract

The transgene‐directed accumulation of non‐native omega‐3 long chain polyunsaturated fatty acids in the seed oil of *Camelina sativa* (Camelina) was evaluated in the field, in distinct geographical and regulatory locations. A construct, DHA2015.1, containing an optimal combination of biosynthetic genes, was selected for experimental field release in the UK, USA and Canada, and the accumulation of eicosapentaenoic acid (EPA) and docosahexaenoic acid (DHA) determined. The occurrence of these fatty acids in different triacylglycerol species was monitored and found to follow a broad trend irrespective of the agricultural environment. This is a clear demonstration of the stability and robust nature of the transgenic trait for omega‐3 long chain polyunsaturated fatty acids in Camelina. Examination of non‐seed tissues for the unintended accumulation of EPA and DHA failed to identify their presence in leaf, stem, flower, anther or capsule shell material, confirming the seed‐specific accumulation of these novel fatty acids. Collectively, these data confirm the promise of GM plant‐based sources of so‐called omega‐3 fish oils as a sustainable replacement for oceanically derived oils.

## Introduction

There is continued interest in the sustainable production of omega‐3 long chain polyunsaturated fatty acids (LC‐PUFAs), also known as omega‐3 fish oils, based on their central importance in marine aquaculture and also human health and nutrition (Tocher *et al.*, 2019). One approach, which has successfully gone from theoretical concept to commercial prototyping, is the use of transgenic plants to accumulate these valuable fatty acids in their seed oil (Napier *et al.*, 2018; 2019). In such a scenario, genetic modification (GM) is used to introduce the non‐native biosynthetic pathway for omega‐3 LC‐PUFAs into the nuclear genome of a suitable oilseed host, enabling the plant to convert endogenous C18 fatty acids into the more desirable C20 + LC‐PUFAs such as eicosapentaenoic acid (EPA; 20:5Δ^5,8,11,14,17^) and docosahexaenoic acid (DHA; 22:6Δ^4,7,10,13,16,19^) (Napier *et al.*, 2018). In most cases, this transgenic pathway is encoded by genes originating from marine microalgae (such organisms are the primary producers of omega‐3 LC‐PUFAs), with their expression in the plant restricted to the seed (Petrie and Singh, 2011). By this method, several groups have demonstrated the feasibility of making significant amounts of EPA and/or DHA in the seed oils of both model plant species such as Arabidopsis (Petrie *et al.*, 2012; Ruiz‐Lopez *et al.*, 2013), but also (to varying levels) in oilseed crops such as Linseed, Camelina and Canola (Abbadi *et al.*, 2004; Petrie *et al.*, 2014; Ruiz‐Lopez *et al.*, 2014; Walsh *et al.*, 2016). Very recently, two different transgenic canola lines accumulating omega‐3 LC‐PUFAs have been granted deregulated status in the USA (meaning that they are approved to be grown commercially), also representing the first examples of GM crops with nutritional enhancement traits (reviewed in Napier *et al.*, 2018, 2019). However, some fundamental questions remain regarding the accumulation and compartmentation of EPA and DHA in seed storage lipid.

In particular, although the primary biosynthetic pathway for the synthesis of EPA and DHA is well‐documented (Napier *et al.*, 2015), via the heterologous characterization of desaturase and elongase genes in yeast and plants, the critical contribution of endogenous enzyme activities, especially in the post‐synthesis accumulation and compartmentation of the omega‐3 LC‐PUFAs into triacylglycerol (TAG; the predominant storage lipid in seed oils), is significantly less well‐understood. For example, the TAG biosynthetic pathways by which EPA and DHA are removed from the metabolic pools, which represent their sites of synthesis (either the acyl‐CoA pool or phospholipids) are known, but the importance of any one route is undefined (and likely to vary between plant species) (Bates, 2016). Gene knockout and overexpression studies in Arabidopsis have identified a number of important enzymes, predominantly acyltransferases, which play roles in acyl exchange and acyl editing, though the metabolic configuration and kinetics of flux through such activities is unknown, either for endogenous or non‐native transgene‐derived fatty acids. Equally, the impact of differing environmental conditions is well‐recognized as altering not only phospholipid acyl‐composition, but also the profile of neutral lipids including TAGs (Rochester and Silver, 1983; Karki and Bates, 2018).

We wished to expand on our previous work demonstrating Camelina as an attractive chassis for lipid metabolic engineering, in particular to better understand omega‐3 LC‐PUFA biosynthesis and accumulation under variable, real‐world conditions. Previous small‐scale pilot studies of field release of GM camelina demonstrated stability of this trait (Usher *et al.*, 2015, 2017), but nothing is known about the impact of different environments. We analysed individual TAG species from one GM Camelina line grown in three different field environments (in the UK, USA and Canada) for two successive years. The large volumes of data generated by this work will ultimately enable us to target those specific biochemical activities likely to either play a key role in oil synthesis or represent points that are sensitive to environmental factors such as variation in abiotic conditions (e.g. temperature; Higashi and Saito, 2019). Such information will be vital to refining our understanding of plant lipid metabolism and to build species‐specific *in silico* models, facilitating the move to truly predictive biology.

## Results and discussion

**Figure 1:**
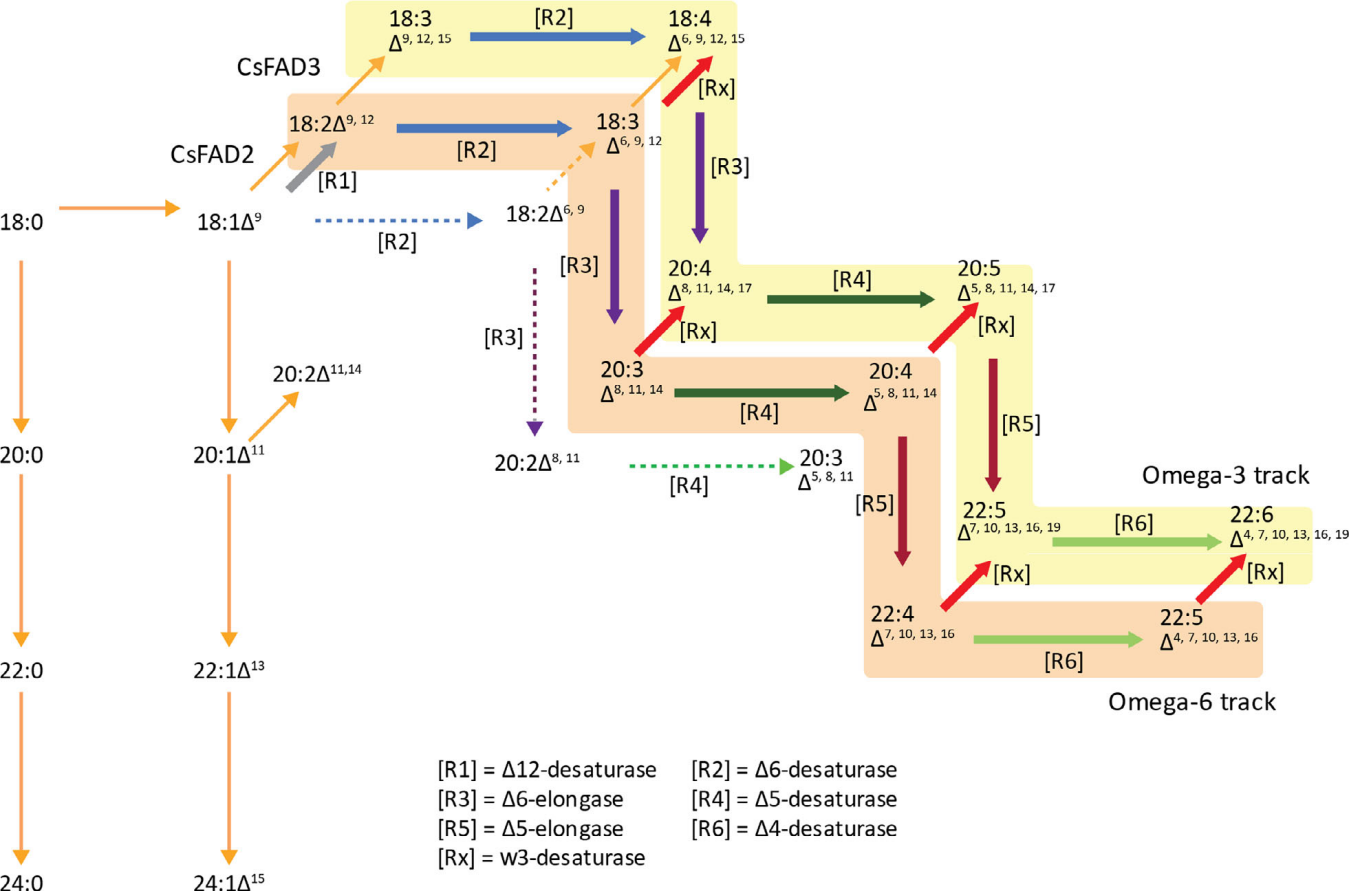
Schematic representation of omega‐3 LC‐PUFA biosynthetic pathway. The enzymatic conversion of fatty acids and the various routes for substrate flux are indicated. The different transgene‐encoded enzyme activities are represented by the coloured arrows and indicated in the figure. The colour coding is maintained in Figure 2a.

**Figure 2:**
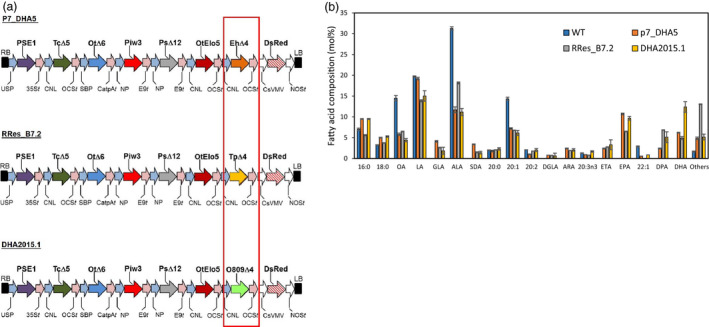
Schematic representation of different constructs used in this study and their efficiency at directing the seed‐specific accumulation of EPA and DHA. (a) Schematic representation of three different constructs (p7_DHA5, RRes_B7.2, DHA2015.1) used to direct the synthesis of EPA and DHA. Different enzyme activities (colour‐coded following the schema used in Figure 1) encoded by synthetic genes under the control of seed‐specific promoters were assembled into binary vectors as described and introduced into *C. sativa* cv. Celine. Abbreviations: CNL, conlinin 1 promoter for the gene encoding the *L. usitatissimum* 2S storage protein conlinin; USP, promoter region of the unknown seed protein of *V. faba*; SBP, sucrose binding protein 1800 promoter; NP, napin; Ot∆6, ∆6‐desaturase from *O. tauri*; Tc∆5, a Δ5‐desaturase from *Thraustochytrium* sp.; Piw3, ω3‐desaturase from *P. infestans*; Ps∆12, a Δ12‐desaturase from *P. sojae*; PSE1, a Δ6‐elongase from *P. patens*; OtElo5, Δ5‐elongase from *O. tauri*; OCS, 35S, E9 and CatpA represent terminators. The varied D4‐desaturase activity is boxed in red, with the EhΔ4, ∆4‐desaturase from *E. huxleyi* being replaced with either TpD4, ∆4‐desaturase from *T. pseudonana* or O809D4, ∆4‐desaturase from *Ostreococcus* RCC809. (b) GC‐FID analysis of total seed fatty acids from glasshouse‐grown transgenic *C. sativa* plants transformed with individual constructs. The superior performance of the DHA2015.1 construct is manifest by the elevated levels of DHA.

The identification of a preferential combination of sequences encoding all of the activities necessary for omega‐3 LC‐PUFA biosynthetic activities, under the control of seed‐specific promoters, was a crucial step in the successful production of non‐native EPA and DHA in plants (pathway illustrated in Figure 1). This particular set of genes (designated p7_DHA5; Ruiz‐Lopez *et al.*, 2014), when expressed in transgenic Camelina, resulted in the noticeable accumulation of both EPA and DHA, differing from analogous efforts in both Camelina and canola by others, which resulted in either the accumulation of just EPA or DHA (reviewed in Napier *et al.*, 2018; Petrie *et al.*, 2014). And as a potential replacement for fish oil, a substitute that contains both EPA and DHA is likely to have greater utility than one that contains only one of these two key fatty acids present in *bona fide* fish oils (Tocher *et al.*, 2019). As discussed elsewhere, this critical difference in terms of the accumulation of EPA and DHA likely reflects the flux of substrates through the pathway, which in turn is modulated both by expression of the transgene activities, but also their individual processivity rates (Allen *et al.*, 2015; Bates, 2016). In addition, the host plant will also play a major determining role, both in terms of the well‐defined levels of substrate fatty acids present in the seed, but also through the opaquer contribution of endogenous activities which facilitate the efficient heterologous reconstitution of omega‐3 LC‐PUFA biosynthesis in plants (Haslam *et al.*, 2016). Such variation will also be modulated by environmental and management factors (Righini *et al.*, 2019). In an effort to further improve our constructs through systematic iteration, efforts were undertaken to identify superior examples of the gene encoding the last step in the omega‐3 LC‐PUFA biosynthetic pathway (Δ4‐desaturase; see Figure 1), since previous studies in Arabidopsis had indicated a role for this enzyme in determining flux through the heterologous pathway and onwards into TAG (Ruiz‐Lopez *et al*, 2013). The Δ4‐desaturase activity from *Emiliania huxleyi* present in p7_DHA5 was therefore replaced with examples from either *Thalassiosira pseudonana* or *Ostreococcus* sp. RCC809, whilst retaining the same regulatory (promoter, terminator) elements, as well as keeping unchanged all other activities present in the original p7_DHA5 construct; Figure 2a). Interestingly, although these three Δ4‐desaturases showed low activity in yeast (Tonon et al., 2005; Vaezi et al., 2013), activities either in their native host (i.e. marine microalga; Jónasdóttir, 2019) or in transgenic plants were significantly higher, emphasizing the ‘context‐dependent’ nature of these enzymes and pathway. They are also quite structurally diverged, showing very limited (<30%) sequence identity (Figure S1). It is noteworthy that the *T. pseudonana* Δ4‐desaturase present in B7.2 was less efficient at converting docosapentaenoic acid (DPA; 22:5Δ^7,10,13,16,19^) to DHA, as indicated by the accumulation of the former, compared with either p7_DHA5 or DHA2015.1. Based on multiple independent transgenic events, it was observed that the construct containing the *Ostreococcus* sp. RCC809 D4‐desaturase (named DHA2015.1) represented a significant improvement on p7_DHA5 (*E. huxleyi* Δ4‐desaturase) and B7.2 (*T. pseudonana *Δ4‐desaturase), resulting in combined EPA and DHA levels routinely in excess of 20% total fatty acids, against the benchmark of 12‐15% for p7_DHA5 (Figure 2b). For that reason, it was decided to proceed with a multinational field evaluation of this most promising line.

### Field evaluation in different environments

Having demonstrated that our new iteration DHA2015.1 was superior, in terms of accumulation of EPA and DHA, to previous combinations of genes, we sought the appropriate regulatory approvals to allow us to undertake field (environmental) releases at different geographical locations (UK, USA and Canada, Table S1). These locations were selected based on a number of different factors including longitude and latitude, local climatic conditions and ease of stewardship and regulatory compliance (Table S2). Thus, approval for experimental GM field release of our DHA2015.1 line was sought and obtained from USDA Animal and Plant Health Inspection Service (APHIS) (for release on the University of Nebraska Experimental Farm, Lincoln, Nebraska, USA) and the Canadian Food Inspection Agency (CFIA) (for release on the AgQuest experimental farm, Elm Creek, Manitoba, Canada) – this was in addition to the pre‐existing approval (16/R8/01) granted by DEFRA (UK) to carry out a field release of DHA2015.1 at the Rothamsted Experimental Farm, Harpenden, UK. Similarly, the appropriate approvals were obtained for the import and movement of these GM seeds within either the US or Canada. Data from a pilot trial release at Harpenden in 2016 confirmed the viability of DHA2015.1 and provided preliminary data as to the performance of the plants in the field, compared with the same line being grown under glasshouse (GH) conditions at the same location (Figure S2). Appropriate sites were prepared for the sowing of these seeds, and the sowing dates were duly recorded (Table S3). Plants were managed according to local experience and crop requirements and grown to maturity prior to harvest. In the case of the UNL (USA) trial, deteriorating weather conditions (including the forecast of tornados) dictated that the crop be harvested prematurely, the impact of which is discussed below.

### Seed fatty acid composition

**Figure 3:**
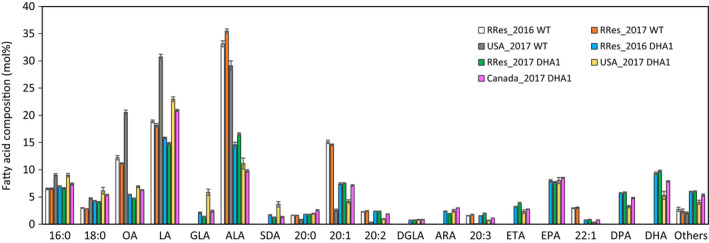
Seed fatty acid composition for all field trials. FAMEs prepared from seed lots samples from the bulked harvests of individual field trials were analysed by GC‐FID, with identification of fatty acids confirmed by GC‐MS and co‐migration with authentic standards. Values are mean ± SE. RRes trials (*n* = 16); USA trial (*n* = 12); Canada trial (*n* = 4).

Fatty acid methyl esters (FAMEs) were prepared from mature seed samples from each GM trial and the associated control (WT Camelina, cv. Celine), and these total FAMEs were resolved and identified by Gas Chromatography‐Flame Ionization Detection (GC‐FID). Multiple technical replicates were analysed for each individual experimental release to provide an average value for seed total fatty acid composition. Data from four different GM field releases are shown in Figure 3 – Rothamsted (UK) 2016 and 2017 (abbreviated to RRes_2016 and RRes_2017), Manitoba 2017 (abbreviated to Canada_2017) and Nebraska 2017 (abbreviated to USA_2017). Some noticeable trends are apparent. Firstly, it is apparent that the engineered EPA + DHA trait in DHA2015.1 is stable under different environments, with the obvious accumulation of both EPA and DHA in all three locations and also in sequential years at the UK site. However, at the same time, whilst the accumulated level of EPA was very similar (~9%) for all four trials, the accumulation of DHA showed greater variation, most noticeably in the case of the USA_2017 trial. As previously noted, this particular trial did not undergo the full seed developmental period and was harvested prematurely. Closer inspection of the seed FAMEs composition of this trial confirms the incomplete developmental programme, indicated by substantially lower levels of 20:1Δ^11^ (a fatty acid normally associated with the accumulation of seed TAGs) and elevated levels of oleic acid (OA; 18:1Δ^9^) and linoleic acid (LA; 18:2Δ^9,12^) in the USA_2017 WT (control) line. Despite this incomplete development, significant levels of omega‐3 LC‐PUFAs still accumulated in the seeds of this GM line.

Some other changes to the seed fatty acid profiles were observed in all releases of DHA2015.1, likely as a combination of the transgenic omega‐3 LC‐PUFA trait and the local environment. Firstly, and in agreement with our previous results in Camelina, it is apparent that α‐linolenic acid (ALA; 18:3Δ^9,12,15^) is the major native fatty acid, which is depleted to facilitate the synthesis of EPA and DHA (Ruiz‐Lopez *et al.*, 2014; Usher *et al.*, 2017). This is in contrast to recent observations in canola, where two very similar efforts to make omega‐3 LC‐PUFAs resulted in seed accumulation of either DHA or EPA but resulted in the predominant depletion of OA and LA (discussed in Napier *et al.*, 2018). As discussed previously, this likely reflects both the differences in how endogenous seed metabolism is configured in Canola versus Camelina, and consequently, the differences in native seed fatty acid composition, as well as the discrete differences in the transgenes (Petrie *et al.*, 2014). However, it is worth noting that some of the enzyme activities present in the EPA‐accumulating Canola line LBFLFK are also present in our Camelina line DHA2015.1, helping to further define the contributions of transgene activities in different native lipid biosynthetic contexts. In the latter case, although OA levels are also impacted by the presence of the transgene pathway (specifically by the presence of a Δ12‐desaturase from the oomycete *Phytophthora sojae*, which converts OA to LA; Lindberg‐Yilmaz *et al.*, 2017), the levels of LA are broadly unchanged in DHA2015.1 compared to WT, irrespective of locations, implying no obvious (substrate/product) relationship between the levels of the two fatty acids (OA, LA) in Camelina. This might (at first hand) appear counter intuitive, given the nature of their biosynthesis (Figure 1), but likely the further metabolism of LA, and the flux through the different enzymes associated with these reactions, can give the impression of static levels of these fatty acids, which almost certainly does not reflect the true metabolic progression of this substrate (as discussed in Bates, 2016). As an illustration, LA is the primary substrate for the transgene‐derived *Ostreococcus tauri *Δ6‐desaturase generating γ‐linolenic acid (GLA; 18:3Δ^6,9,12^), as well as substrate for the endogenous Δ15‐desaturase *FAD3* which converts LA to ALA. In turn, GLA and ALA can be further desaturated to stearidonic acid (SDA; 18:4Δ^6,9,12,15^), with both GLA and SDA serving as substrates for transgene‐derived Δ6‐elongation to C20 forms dihomo‐γ‐linolenic acid (DGLA; 20:3Δ^8,11,14^) and eicosatetraenoic acid (ETA; 20:4Δ^8,11,14,17^) (Figure 1, see also Napier *et al.*, 2015). However, in the absence of tracer studies, it is not possible to determine the contribution of these different routes to the synthesis of downstream products. However, when the trials are ranked for the accumulation of DHA (Figure S3), there is a clear inverse relationship between DHA and LA. For example, the RRes_2016 glasshouse‐grown DHA2015.1 material has the highest level of DHA, but the lowest level of LA; conversely, the Canada_2017 and USA_2017 trials have the second‐lowest and lowest levels of DHA, whereas they show the second highest and highest levels of LA.

It is also worthy of note that the accumulation of biosynthetic intermediates (defined here as any fatty acid in the pathway shown in Figure 1 between endogenous fatty acids LA and ALA and the desired omega‐3 LC‐PUFA products) is relatively modest, especially when compared to EPA and DPA. In the case of these two fatty acids, which still require two (EPA) or one (DPA) further enzymatic modifications to generate the final product DHA, it appears that endogenous factors disproportionately (and serendipitously) direct the accumulation of these intermediates into storage lipid (TAG) as a metabolic dead‐end, whilst simultaneously allowing for a percentage to be further metabolized to DHA. The molecular basis for this discrimination, allowing both flux and accumulation, is not the direct consequence of transgenesis, since no activities involved in acyl exchange between different metabolic pools are present in the DHA2015.1 construct, though it has been proposed that differences between the processivity of individual members of the transgene‐derived biosynthetic pathway can contribute to the enrichment for these end products (Petrie *et al*, 2014). Irrespective of this, there is again very limited difference between the seed FAMEs profiles of plants grown in different locations or years, apart from the already discussed example of the premature harvest of the USA_2017 material. In that example, although EPA levels are similar to those found in the other trials, DPA and DHA levels are notably depressed, meaning that total C20 + omega‐3 LC‐PUFAs were significantly reduced in this one trial. That this is as a consequence of endogenous metabolism, as opposed to transgene‐derived, is evidenced by the fact that the last three reactions in the omega‐3 LC‐PUFA biosynthetic pathway (Δ5‐desaturase, Δ5‐elongase, Δ4‐desatutase; Figures 1 and 2a) are all under the control of the same seed‐specific promoter (CNL), implying the simultaneous transcription of these activities. Moreover, since the Δ5‐desaturase is responsible for the synthesis of EPA from ETA, yet EPA levels are unaffected in the USA_2017 trial, it can be concluded that the regulatory transcription factors which modulate the expression of the CNL promoter were present at this incomplete stage of seed development. Our observations would therefore indicate a temporal aspect of the flux of non‐native fatty acids into TAG, with the incorporation of the C20 EPA preceding that of C22 PUFAs such as DPA and DHA. This would echo the observations of Pollard *et al *(2015) who noted a strong time‐dependent incorporation of ALA into TAG, at the expense of other abundant fatty acids such as LA, OA and 20:1Δ^11^. The biochemical basis for such disparity in the temporal accumulation of EPA versus DHA is unknown, but may reflect developmental regulation of different routes into TAG, such as diacylglycerol acyltransferase (DGAT), phospholipid:diacylglycerol acyltransferase (PDAT) and phosphatidylcholine‐diacylgycerol (PC‐DAG) acyl exchange.

### Seed TAG composition

**Figure 4:**
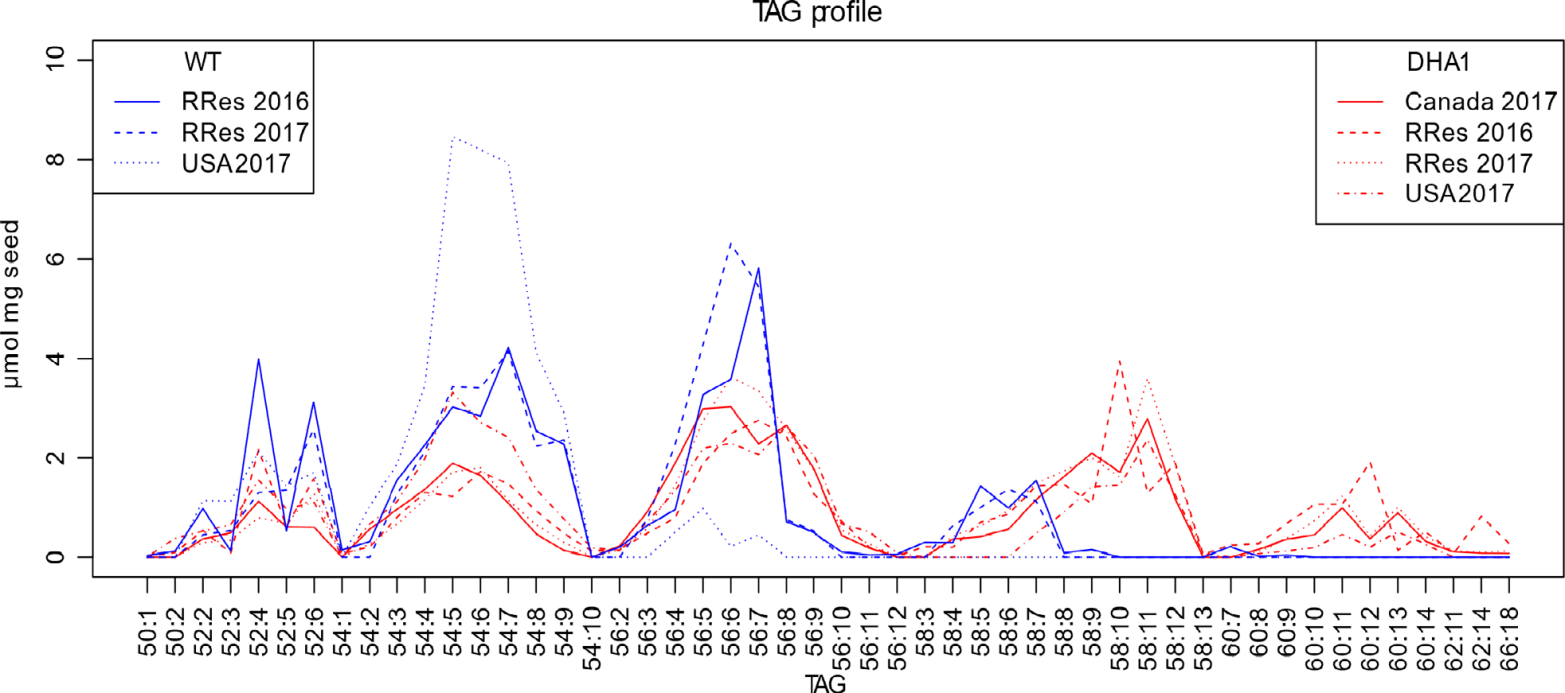
Analysis of triacylglycerols from *C. sativa* seeds. Individual TAG molecular species from transgenic or control lines were resolved by HPLC and identified by ESI‐MS/MS neutral loss survey scan with each TAG species represented by the total number offatty acid carbon atoms:desaturations, as previously described (Usher *et al.*, 2017). 100 mg of seed per replicate; *n* = 16 except for USA 2017 WT (*n* = 12), USA 2017 DHA1 (*n* = 11) and DHA1 Canada 2017 (*n* = 6).

Acyl‐composition of seed storage oil was determined by electrospray ionization mass spectrometry (ESI‐MS/MS), revealing a diversity of individual TAG species (53 different configurations in WT, 98 in DHA2015.1, including tri‐DHA), which reflected the differences in FAMEs described above. The most marked differences could be attributed to the transgene‐dependent presence of the non‐native LC‐PUFAs such as EPA and DHA, as well as the associated decrease in ALA and 20:1Δ^11^. This is most clearly seen with the presence of TAG species C58:8+ (i.e. TAG molecules in which the total acyl carbons were 58 and containing 8 or more double bonds), indicated with red lines (Figures 4 and S4). Given that these C58:8 + TAGs are only present in the DHA2015.1 line and using the seed FAMEs data (Figure 3) as a reference, they likely comprise at least one EPA (20:5) and one LA (18:2) and one 20:1Δ^11^, although different permutations can be envisaged. Similarly, the C60 + TAGs, which are only present in DHA2015.1 (to varying levels, presumably in an environment‐dependent fashion), most likely contain either two EPA, or an EPA and DHA, molecules. As discussed above, the total seed fatty acid profile in DHA2015.1 is not only altered by the presence of non‐native omega‐3 LC‐PUFAs, but also by a reduction in two endogenous fatty acids ALA and 20:1Δ^11^. Interestingly, this has a broad impact on multiple TAG species, indicating (as would be expected) that these two fatty acids are incorporated into many different TAG configurations. This is particularly striking in the case of the C54:X TAGs, which are likely comprised of three C18 fatty acids (or 16 + 18+20) (Figure S5). Since the accumulation of ALA is notably reduced in DHA2015.1, and this particular fatty acid is known to be abundant in TAG of WT Camelina (Pollard *et al.*, 2015), it is not surprising that there is a concomitant perturbation to the seed oil profile. It is also of note that the TAG species of DHA2015.1, irrespective of environment or generation, shows a broadly similar modified profile to that reported for p7_DHA5 (Usher *et al.*, 2017).

A principal component analysis (PCA) on the TAG composition of WT and DHA2015.1 reveals a distinct clustering in samples taken from the same field condition on the first two principal components (Figure S4). The primary distinguishing feature is the separation of WT and transgenic lines, based on the TAG carbon number (e.g. 52:x and 54:x TAG in WT; 56:x to 60:x TAG in DHA2015.1). The impact of year and location is specifically seen in the WT TAG data, and RRes_2017 and RRes_2016 have different 52:x and 54:x TAG species. The USA WT trial clusters away from both RRes WT trials. The DHA2015.1 trials, however, show much less variation with year and location (only USA_2017 clusters apart), suggesting that the synthesis on non‐native EPA and DHA is not significantly impacted by year or location (Figure S4). Although there is evidence of local adaptation in the seed oil TAG composition; the omega‐3 trait is stable, reflecting field seed FAMEs data.

### Agronomic performance

**Figure 5:**
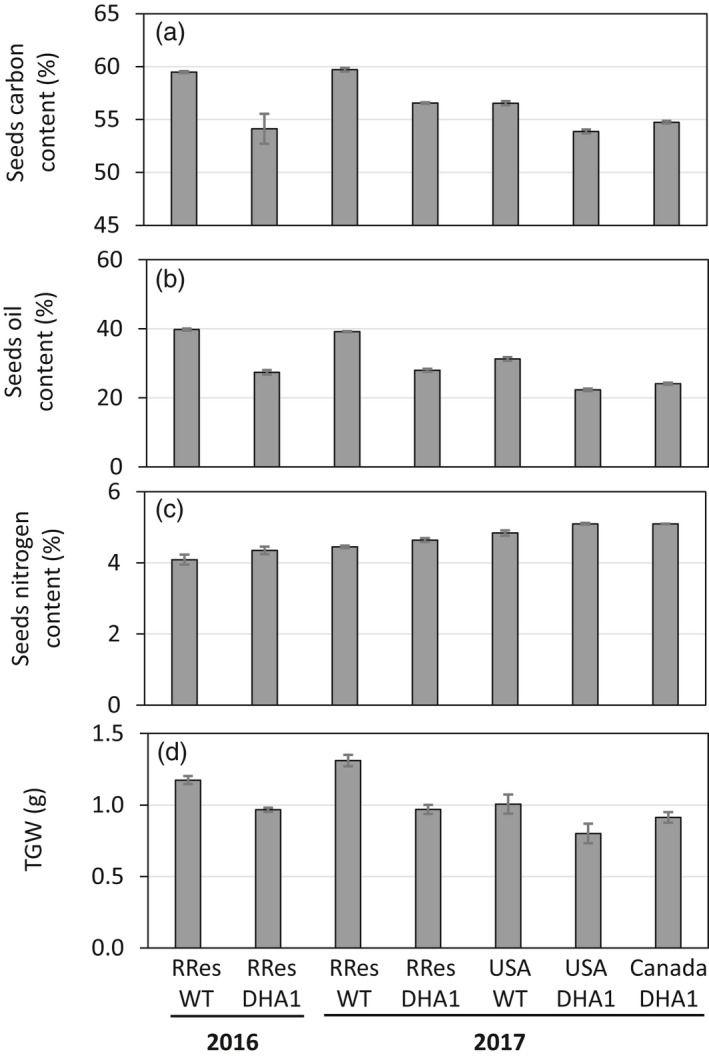
Agronomic performance. (a) Total seed carbon; (b) Oil content; (c) Total seed nitrogen and (d) thousand grain weight were determined and presented for each field trial of WT and GM *C. sativa*. Values are mean ± SE. Carbon content (*n* = 6 or 8); Oil content (*n* = 3 or 4); TGW (*n* = 3 or 4).

Previous analysis of the field‐grown line p7_DHA5 (Usher *et al.*, 2015, 2017) indicated that this transgenic line had some minor yet noticeable alterations to their seed compositions. To provide further data and insights into the possible nature of these changes, similar analyses were carried out on all the DHA2015.1 field releases described in this study – by this approach, we hoped to gain an insight into the contribution of environmental factors to these perturbations. As shown in Figure 5a,b, and as previously observed by Usher *et al. *(2017), there was a clear inverse correlation between the accumulation of omega‐3 C20 + LC‐PUFAs and both total seed carbon and seed oil content. Markedly, the higher the accumulated levels of EPA, DPA and DHA, the stronger the apparent repression of seed oil synthesis and concomitant reduction in total seed oil content. This phenomenon has been previously observed not only for LC‐PUFAs (Petrie *et al.*, 2014), but also for other non‐native fatty acids such as ricinoleic acid with the mechanism by which this so‐called ‘oil yield penalty’ occurs believed to be via the repression of plastidial fatty acid synthesis (FAS) (Bates *et al.*, 2014) and can be partially rescued by overexpression of the WRINKLED1 (WRI1) regulatory factor (Adhikari *et al*, 2016). The data from our multilocation field trials provide several new insights into this overall process. Firstly, the repression of seed oil content is predominantly as a consequence of the transgene‐derived metabolic changes to lipid metabolism, but there is also clearly a contribution from environmental factors, as evidenced by the variation in the degree of repression observed. Secondly, in the case of the developmentally incomplete USA trial, the oil yield penalty is already manifest, and to the same magnitude as in the UK trials, even though the seeds have not undergone the full development and maturation process. This implies that the initiation of repression of seed oil synthesis in DHA2015.1 occurs concomitant with the synthesis and accumulation of non‐native fatty acids such as EPA and DHA, most likely at mid‐stage of seed development as defined by Pollard *et al. *(2015) and Abdullah *et al. *(2016).

Beyond the impact on seed oil content, several other agronomic measures indicated differences either due to transgenesis or location. For example, total seed nitrogen levels (as a percentage of total dry matter) were slightly elevated in the DHA2015.1 event, in all locations (Figure 5c). This is in agreement with previous observations (Usher *et al.*, 2017), although the basis for this is unknown. A plausible explanation might be that this represents a rebalancing of seed composition, reflecting the long‐established inverse relationship between seed protein and seed oil content, although the magnitude of the changes does not directly support this. Perhaps easier to explain, both the thousand grain weight (TGW) (Figure 5d) and total seed carbon content display very similar magnitudes of change as a consequence of the DHA2015.1 transgenesis, showing marked reduction in both measurements. This likely reflects the reduction in total seed oil, which is the major store for seed carbon and also determinant for seed weight. Again, a similar trend was observed for the previous iteration, line p7_DHA5 (Figure 2a) when grown under field conditions on the Rothamsted farm (Usher *et al.*, 2017) – see also Table S4 for statistical consideration of these data.

### Tissue‐specific accumulation of EPA and DHA

During our field trials, a report was published of a small‐scale laboratory study in which Cabbage White *Pieris rapae* caterpillars were feed an artificial diet containing EPA and DHA, apparently resulting in developmental defects in the adult butterflies (Hixson *et al.*, 2016). The same authors subsequently speculated that GM crops accumulating EPA and DHA might have serious unintended impacts on terrestrial ecosystems, through the proposed toxic impact of these fatty acids accumulating in vegetative tissues consumed by herbivorous insects and called for greater regulatory oversight on the field release and commercialization of such crops (MacDonald *et al.*, 2018). Although the experimental observations of Hixson *et al. *(2016) were obtained from a single experiment and were based on the ingestion of EPA and DHA as free fatty acids (FFAs) as opposed to TAGs (the primary lipid accumulating EPA and DHA in our transgenic plants), we sought to determine if any omega‐3 LC‐PUFAs accumulated in non‐target tissues of the transgenic DHA2015.1 Camelina plant. This was in part as an attempt to address the statement in Hixson *et al *(2016) ‘*It should be noted that EPA and DHA biosynthesis may be controlled by seed‐specific promoters […], and therefore the transgene may be expressed in the seed only; however, the absence of EPA and DHA in other plant tissues has yet to be confirmed*’ and also to provide data as to the ‘real‐world’ performance and absolute specificity of the various seed‐specific promoters used in the DHA2015.1 construct.

**Figure 6:**
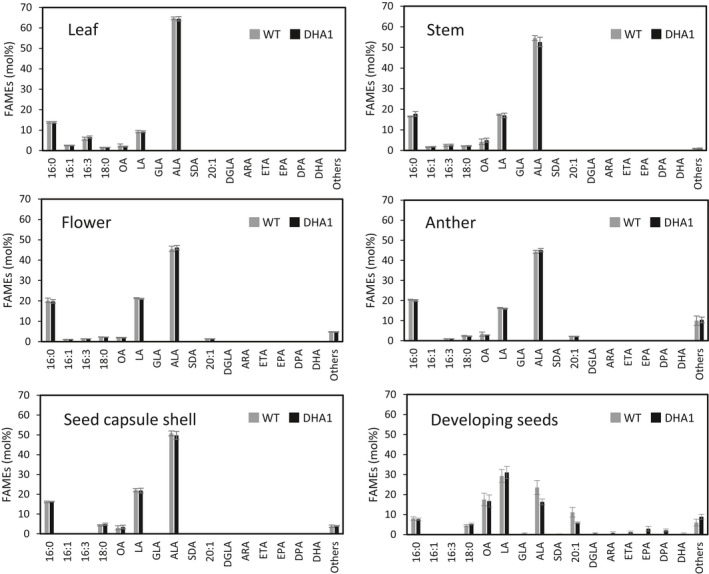
Distribution of EPA and DHA in non‐seed tissues. Leaves, stems, flowers, anthers, seed capsule shells and developing seeds samples were collected from 2017 Rothamsted field trial at approximately 15–18 days after flowering based on visual inspection. FAMEs were prepared from tissue samples either the DHA2015.1 block or the WT control and analysed by GC‐FID, with identification of fatty acids confirmed by GC‐MS and co‐migration with authentic standards. Values are mean ± SE (*n* = 8).

Tissue samples were taken from the leaves, stems, flowers, anthers, seed capsule shells and developing seeds of DHA2015.1 and WT camelina plants grown at Rothamsted in 2017 and used to prepare FAMEs for GC‐FID and GC‐MS analysis. In the case of the green seed capsules, these were split open and the developing seeds removed and analysed separately. As shown in Figure 6, the fatty acid profiles for all tissues apart from developing seeds are devoid of EPA and DHA, or any other biosynthetic intermediate on the LC‐PUFA biosynthetic pathway (such as GLA or SDA – Figure 1). In the case of developing seeds, C20 + LC‐PUFAs were clearly present to varying levels, commensurate with the recent initiation of biosynthesis and as would be expected for transgenes driven by seed‐specific promotors such as napin (NP) from *Brassica napus*, conlinin (CNL) from *Linum usitatissimum* and unknown seed protein (USP) from *Vicia faba*. That these seeds have only partially completed the development phase is indicated by the rank‐ordered relative abundance of seed fatty acids, with LA the most abundant (LA > ALA > OA > 20:1) (Abdullah *et al.*, 2016; Pollard *et al.*, 2015). Collectively, our data confirm that the seed‐specific promotors used in our study restrict the accumulation of non‐native omega‐3 LC‐PUFAs to the seed, and no ectopic accumulation of EPA and DHA was detected in non‐seed tissue. This included anthers, in which some seed‐specific promoters have been shown to have activity in the pollen (Zakharov *et al.*, 2004). Thus, the suggestion that GM plants engineered to accumulate EPA and DHA in a seed‐specific manner may also accumulate these fatty acids in other tissues (through misexpression of the biosynthetic transgenes) is not proven. This agrees with a recent proteomic study demonstrating that transgene‐derived proteins of the omega‐3 LC‐PUFA biosynthetic pathway in canola were only detected in seeds but no other tissues (Colgrave *et al.*, 2019).

## Conclusions

A number of potentially relevant conclusions can be drawn from this study. Firstly, although we only made a single modification to our construct (swapping the *Emiliana huxleyi* Δ4‐desaturase for a similar activity from *Ostreococcus* RCC809), this had a pronounced effect on the total seed fatty acid composition and to a lesser extent, the seed TAG profile (Usher *et al.*, 2017). That changing the final enzyme activity in a biosynthetic pathway can result in such noticeable differences might at first appear counterintuitive, but this can be explained by several factors. Firstly, although both the *E. huxleyi* and *Ostreococcus* RCC809 Δ4‐desaturases have been demonstrated to be active in heterologous systems, nothing is known about their enzyme kinetics or contribution to flux through the omega‐3 biosynthetic pathway. Both desaturases are assumed to use phosphatidylcholine (PC)‐linked substrates, as opposed to acyl‐CoA substrates, based on *in vitro* studies of related sequences (Lindberg Yilmaz *et al.*, 2017; Figure S1), implying that DHA is generated on PC and must be removed from this site of synthesis to the final metabolic destination of TAG, most likely by the acyl‐CoA‐independent activity of PDAT (see Figure S6). Perhaps less obvious is how different amino acid sequences which encode the same desaturase activity can generate differences in total seed FAMEs (e.g. see Figure 2b for three different Δ4‐desaturase activities). Certainly, one likely factor is the ‘interactomes’ generated by these individual enzymes (Coleman, 2019), which are likely to depend on protein–protein interactions mediated by secondary and tertiary structures generated by apparently minor variations in the primary amino acid sequences. This is an emerging topic in the study of plant pathways, but it is interesting to note that previous genetic studies identified a protein (PAS1) proposed to act as a protein scaffold in the assembly of the microsomal fatty acid elongation complex (Roudier *et al.*, 2010). These interactomes may also serve as metabolons, helping to channel‐specific substrates to appropriate enzymes.

A second important conclusion from this study is the demonstration of the stability of the omega‐3 LC‐PUFA trait in the field under real‐word conditions. This is relevant given the significant interest in developing viable alternatives to the wild‐capture of fish from our oceans (with all the attendant environmental impacts and sustainability issues) as a source of these valuable fatty acids (Tocher *et al.*, 2019). Very recently, two companies have developed canola lines which have been engineered to accumulated omega‐3 LC‐PUFAs (reviewed in Napier *et al.*, 2018) though these Canola events differ from the Camelina line described in this study, in that they predominantly accumulate either EPA or DHA. In the case of the DHA2015.1 Camelina line described here, the transgene‐mediated metabolic engineering results in the synthesis and accumulation of both DHA and EPA, and in that respect, it is an important demonstration that a trait for the combination of both these fatty acids is also stable in different environments. The concomitant presence of EPA and DHA is also an important consideration in the commercial viability of GM camelina, compared with the above‐mentioned Canola products, since it is well‐established that the overall seed oil yield of camelina is lower (at ~800 kg/ha) than that observed for Canola (~1000 kg/ha) (Krzyżaniak *et al.*, 2019). In the case of Camelina lines accumulating EPA and DHA, this seed oil content could be further reduced through the manifestation of the oil yield penalty (estimated 70% of WT seed oil levels; Figure 5b) to ~560 kg/ha. However, if the amount of omega‐3 LC‐PUFAs (EPA, DPA and DHA) is calculated as a percentage of these oil yield, then Camelina (at 23.3% of total fatty acids) is superior to Canola (11.1%), with 130.5Kg (Figure S3, data for RRes 2017) as opposed to 111 kg for Canola (Napier *et al.*, 2019). In fact, these are likely to be ‘worst‐case’ figures, since data on the oil yield penalty in the omega‐3 Canola are missing and also agronomic management of our Camelina was sub‐optimal (no weed control, low nitrogen fertilization). Collectively, these calculations show great promise for GM Camelina accumulating EPA and DHA as an economically viable replacement for oceanically derived fish oils. Perhaps more pertinently, and of relevance also to some of the algal products which are rich in only DHA (Tocher *et al.*, 2019), only our GM camelina has an omega‐3 LC‐PUFA profile which is closely matched with the original product it aims to replace.

The (likely synergistic) contribution of transgene‐derived activities and endogenous metabolism is also apparent from comparison between the performance of DHA2015.1 construct and different configurations of the same pathway by others. For example, Petrie *et al. *(2014) introduced the same activities (but encoded by different genes) present in DHA2015.1 (Figures 1 and 2a) into transgenic Camelina, also under the control of seed‐specific promoters. However, their GA7 construct in Camelina generated not only DHA as previously observed in Arabidopsis (Petrie *et al.*, 2012), but also elevated levels of the intermediate SDA. The basis for the atypical accumulation of SDA in the GA7 DHA camelina is not clear, but likely reflects the ‘trapping’ of SDA in a metabolic impasse, most likely TAG, as a consequence of the acyl‐CoA‐dependent Δ6‐desaturase generating SDA‐CoA which is then used as a substrate by acyltransferases such as DGAT. Equally, the production of DHA in the GA7 Camelina was at the expense of LA but not ALA, unlike observed by us here and previously (Petrie *et al.*, 2014; Ruiz‐Lopez *et al.*, 2014). This indicates the interplay between the context‐dependent nature of metabolic engineering for the heterologous synthesis of omega‐3 LC‐PUFAs and also the importance of selecting the best configuration of efficient transgene activities (Haslam *et al.*, 2016).

By way of an example, in analogous efforts by others, transgenic canola was engineered with a set of genes very similar to those used in DHA2015.1, differing only in the presence of additional sequences for several activities (reviewed with full description in Napier *et al.*, 2018), as well as lacking the superior *Ostreococcus* RCC809 Δ4‐desaturase described here. In that configuration and context, the fatty acid profile of the transgenic Canola was biased towards the accumulation of EPA at the expense of DHA, despite using the ELO5 Δ5‐elongase activity from *Ostreococcus tauri* also present in DHA2015.1. In that respect, the ratio of EPA:DPA:DHA in Canola line LBFLFK (Napier *et al.*, 2018) resembles the incomplete seed development of the USA_2017 trial, although the fatty acid composition of this Canola event was tested at multiple locations and occasions, ruling out local environmental factors. Of note, the regulatory elements controlling the expression OtElo5 differ between DHA2015.1 and LBFLFK, with the former being under the *L. usitatissimum* conlinin promoter and the later using the *Brassica napus* FAE1.1 promoter. Conlinin is a seed‐storage protein promoter and is known to be active in mid‐stage of seed development, whereas FAE1 controls the expression of the 3‐ketoacyl CoA synthase (KCS) activity responsible for the synthesis of 20:1Δ^11^, which occurs at a slightly later point in seed development, and they do not completely share common transcription factors. For optimal accumulation of EPA and DHA, it may make sense to ensure the co‐ordinated expression of transgene‐encoded biosynthetic activities.

### Making sense of the process – what does it all mean?

As our understanding of the complexity of metabolism grows, along with a better appreciation of the exquisite regulation with which seed development is subject to, it can sometimes seem almost surprising that the transgenic (partial) addition of a heterologous pathway can result in the successful reconstitution of omega‐3 LC‐PUFA synthesis. The reliance on endogenous acyltransferase enzymes to shuttle non‐native fatty acids between the two metabolic hubs of PC and acyl‐CoAs might be predicted to generate a bottleneck that stalls this pathway. In fact, such a blockade has previously been observed in other plant species (Abaddi *et al.*, 2004) and it may be that Camelina as a hexaploid simply contains more genetic variation in these key endogenous enzymes, facilitating the flux of substrates between these two pools (Malik *et al.*, 2018). As described above, recent attempts to engineer canola to accumulate EPA and DHA have notably not been as successful as we have observed for camelina (Napier *et al.*, 2018), emphasizing the species‐specific nature of the metabolic context into which the transgene‐derived activities need to operate. Equally, our earlier attempts to engineer Arabidopsis with the capacity to synthesis omega‐3 LC‐PUFAs via expression of the p7_DHA5 cassette resulted in low (<8%) levels of EPA and DHA (Ruiz‐Lopez *et al.*, 2013) – cf. Figure 2b in this study. It is likely that each plant species has a different configuration of endogenous lipid metabolism, as a consequence of the sum of multiple small variations in both the regulation and substrate specificity of the enzymes which contribute to this process. This helps explain the unsuccessful search for a single ‘magic bullet’ which directs the high level accumulation of non‐native fatty acids – instead, there are many examples of transgenic manipulations resulting in incremental gains in target fatty acid accumulation, mediated by different enzymes (DGAT, PDAT – Bates et al., 2014; phosphatidylcholine diacylglycerol cholinephosphotransferase (PDCT) – Yu *et al.*, 2019; phospholipase‐C (PLD‐C) – Aryal *et al.*, 2018; PLD‐D – Yang *et al.*, 2017). Collectively, these observations make the case for a better understanding of metabolic flux in individual plant species, as well as bespoke genetic interventions to maximize the accumulation of target fatty acids based on the very best understanding of the biochemical processes which underpin these processes (Figures 1 and S6) (Haslam *et al.*, 2016; Sweetlove *et al.*, 2017). It is very likely that flux (which is not determined in steady‐state analyses of total fatty acids present here) is equally as important a criterion for the identification of optimal combinations of transgene‐derived enzyme activities (Bates, 2016).

One final consideration is the need for integration of both lipidomic and transcriptomic datasets, enabling the development of better, testable, models of these pathways (Abdullah *et al.*, 2016). Ultimately, these will also need to incorporate the spatial heterogeneity we and others have observed in seed metabolism (Horn and Chapman, 2014; Lu *et al.*, 2018; Marmon *et al.*, 2017; Usher *et al.*, 2017), which could help to lead to the goal of predictive manipulation of plant seed composition.

## Materials and methods

### Plant material and growth conditions

*Camelina sativa* (cv. Celine) was used in all experiments. Plants grown in the glasshouse were maintained in controlled conditions at 23°C day/18°C night, 50–60% humidity and kept under a 16‐h photoperiod (long day), with supplemental light provided when ambient levels fell below 400 µmol/m^2^/s. Harvest usually occurred 100 days after sowing. A summary of the environmental conditions at all three trial sites is shown in Tables S2 and S3.

### Generation of transgenic plants

Transgenic *C. sativa* lines were generated as previously described (Ruiz‐Lopez *et al.*, 2014). The designed vectors were transferred into *Agrobacterium tumefaciens* strain AGL1. *C. sativa* inflorescences were immersed in the *Agrobacterium* suspension for 1 min without applying any vacuum. Transgenic seeds expressing the EPA and DHA pathway were identified by visual screening for DsRed activity. Seeds harvested from transformed plants were illuminated using a green LED light. Fluorescent seeds were visualized using a red lens filter.

### Vector construction

Three constructs, as described, containing cassettes of seven genes (p7_DHA5, RRes_B7_2, DHA2015.1; Figure 2a) were used for plant transformation. The p7_DHA5 has been previously described (Ruiz‐Lopez *et al.*, 2014). All three constructs contained a Δ6‐desaturase gene from *O. tauri* (OtΔ6), a Δ6 fatty acid elongase gene from *Physcomitrella patens* (PSE1), a Δ5‐desaturase gene from *Thraustochytrium* sp. (TcΔ5), a Δ12‐desaturase gene from *Phytophthora sojae* (PsΔ12), an ω3‐desaturase from *Phytophthora infestans* (Piw3) and an *O. tauri* Δ5 fatty acid elongase gene (OtElo5). The only difference between the three constructs was as a consequence of varying the Δ4‐desaturase gene. Thus, in p7_DHA5 this activity was from *Emiliania huxleyi* (EhΔ4), in RRes_B7.2 is was from *Thalliosira pseudonana* (TpD4) and in DHA2015.1 it was from *Ostreococcus* RCC809 (O809D4). All open reading frames for desaturases and elongases were re‐synthesized (GenScript Corporation, NJ, www.genscript.com) and codon optimized for expression in *Arabidopsis thaliana*. All genes were individually cloned under the control of seed‐specific promoters and then combined into a single T‐DNA transformation vector as previously described (Ruiz Lopez *et al.*, 2014). The destination binary vector contained a DsRed marker within the T‐DNA sequence for visual selection of GM plants.

### Field trials

Field experiments conducted at Rothamsted Research in 2016 and 2017 (Harpenden, Hertfordshire, U.K.; grid reference TL120130) were carried out as previously described (Usher *et al.*, 2015, 2017), under DEFRA consent 16/R8/01. Field trials in Canada were managed by Ag‐Quest (Minto, Manitoba; https://agquest.com) including all aspects of approvals from CFIA for environmental release. Similarly, field trials in USA were managed by University of Nebraska, Lincoln experimental farm facility, part of the Department of Agriculture and Horticulture, including obtaining approvals from APHIS for environmental release. The detailed sowing dates and locations of the GM field trials are described in the Supplementary data (Table S1). Unless stated otherwise, for all the experimental data analysis, the values of each Camelina line were given as mean value ± standard error from each line replicate plots.

### Assessment of agronomic performance

Total carbon and nitrogen content were determined by combustion using a Combustion Analyser (LECO TruMac, LecoCorp, St.Paul, MN). This was performed by the in‐house analytical unit at Rothamsted Research. Data are present as a percentage of 100% dry matter content. Two replicate samples were collected from each plot. Total seed oil was measured by NMR. Each seed sample (about 2g) is placed into the NMR tube, weighted and measured and then calculated the oil content according to the calibration curve. Thousand grain weight is measured by weighing 1000 dry seeds. For seed oil and TGW analysis, one sample is collected from each plot. Technical replicates were then drawn from this single sample.

### Fatty acid analysis

Total fatty acids in seed batches were extracted and transmethylated according to previous methods (Ruiz‐Lopez *et al.*, 2014). Four biological replicates were sampled from each plot, with the amount of 100mg dry seeds each replicate. Methyl ester derivatives of total fatty acids extracted were analysed by Gas Chromatography‐FID (flame ionization detection), and the results were confirmed by GC–MS. Minor fatty acids (such as 16:1n‐7, 18:2trans, 20:1n‐7, 20:2trans, 22:0, 22:2n‐6 and 24:0) were summed and are presented as *others*.

### Lipid analysis

Triacylglycerols (TAGs) were measured in Camelina seed from seed harvested from the field trial. The sampling method is the same with that of fatty acid analysis. TAGs were measured according to Usher *et al. *(2017) and were defined by the presence of one acyl fragment and the mass/charge of the ion formed from the intact lipid (neutral loss profiling). This allows identification of one TAG acyl species and the total acyl carbons and total number of acyl double bonds in the other two chains. The procedure does not allow identification of the other two fatty acids individually nor the positions (sn‐1, sn‐2, or sn‐3) that individual acyl chains occupy on the glycerol. TAGs were quantified after background subtraction, smoothing, integration, isotope deconvolution and comparison of sample peaks with those of the internal standard (using Lipid‐View^TM^; Sciex). The data were normalized to the internal standards tri15:0 and tri19:0 (Nu‐Chek Prep, Elysian, MN). The profiling samples were prepared by combing 50 uL of the total lipid extract with 950 uL of isopropanol/methanol/50 mm ammonium acetate/dichloromethane (4:3:2:1). Samples were infused at 15 uL/min with an autosampler (CTC‐PAL, CTC Analytics). The scan speed was 100 u/s. The collision energy, with nitrogen in the collision cell, was + 25 V; declustering potential was + 100 V; entrance potential was 14 V; and exit potential was + 14 V. Sixty continuum scans were averaged in the multiple channel analyser mode. For product ion analysis, the first quadrupole mass spectrometer (Q1) was set to select the TAG mass and Q3 for the detection of fragments fragmented by collision induced dissociation. The mass spectral responses of various TAG species are variable, owing to differential ionization of individual molecular TAG species. For all analyses, gas pressure was set on ‘low’, and the mass analysers were adjusted to a resolution of 0.7 L full width height. The source temperature was 100 °C; the interface heater was on, and +5.5 kV was applied to the electrospray capillary; the curtain gas was set at 20 (arbitrary units); and the two ion source gases were set at 45 (arbitrary units). In the data shown herein, no response corrections were applied to the data. The data were normalized to the internal standards tri15:0 and tri19:0 (Nu‐Chek Prep, Elysian, MN).

### Tissue‐specific analysis

Leaves, stems, flowers, anthers, seed capsule shells and developing seeds samples were collected from 2017 Rothamsted field trial at approximately 15–18 days after flowering based on visual inspection according to Rodríguez‐Rodríguez *et al. *(2013). Two replicate samples were collected from each plot. The detailed sampling method was as follows. The entire leaf next to the first branch of the main stem was collected. Stem samples were collected from the main stem next to the sampled leaf, excising 6 cm of material from this junction and towards the roots. Whole newly opened flowers (*n* = 10/sample) were collected for flower lipid analysis. Similarly, anthers were collected from newly opened flowers, with *n* = 10 flowers for each replicate sample. The seed capsules were collected from the third or fourth pods of the main stem (counting up towards the apex), with *n* = 10 for each replicate sample. Capsules were split into developing seeds and residual capsule shells for fatty acid composition analysis.

## Conflict of interest

The authors declare that none of them have a conflict of interest.

## Author contribution

The study was initiated by JAN and OS, and the field studies were planned and carried out by LH with support from SU and SS. Lipid and lipidomic analysis was carried out by LH, LVM and RPH. Statistical analysis was carried out by KH. The manuscript was written by JAN with contributions from all authors.

## Supporting information


**Figure S1 **Sequence line‐up of the three D4‐desaturase sequences tested in this study.
**Figure S2** GC‐FID analysis of FAMEs from the mature seeds of DHA1 plants grown in greenhouse conditions at Rothamsted, compared with the control variety (Celine).
**Figure S3** GC‐FID analysis of FAMEs from the mature seeds of DHA2015.1 plants grown in different field locations, rank‐ordered on the basis of the accumulation of DHA.
**Figure S4** Statistical analysis of field‐grown Camelina seed TAG data.
**Figure S5** Seed TAG profile from replicate analysis of pooled plot samples (100 mg of seed per replicate; *n* = 16 except for WT USA 2017 (*n* = 12), DHA1 USA 2017 (*n* = 11) and DHA1 Canada 2017 (*n* = 6) as determined by ESI‐MS/MS analysis (QTRAP 4000).
**Figure S6** Schematic representation of the Kennedy pathway and the biosynthetic routes to storage lipid (TAG).
**Table S1 **Field trial location.
**Table S2 **Field trial Rothamsted, USA & Canada weather condition.
**Table S3 **Field trial Camelina growth season.
**Table S4 **Statistical comparisons between DHA and WT.
