# Supplementary material for: High level accumulation of EPA and DHA in field‐grown transgenic Camelina – a multi‐territory evaluation of TAG accumulation and heterogeneity
Source: Plant Biotechnol J. 2020 May 8;18(11):2280–91. doi: 10.1111/pbi.13385 (PMC7589388; doi:10.1111/pbi.13385)
Supplement: Supplementary file 1 — Figure S1 Sequence line‐up of the three D4‐desaturase sequences tested in this study. Figure S2 GC‐FID analysis of FAMEs from the mature seeds of DHA1 plants grown in greenhouse conditions at Rothamsted, compared with the control variety (Celine). Figure S3 GC‐FID analysis of FAMEs from the mature seeds of DHA2015.1 plants grown in different field locations, rank‐ordered on the basis of the accumulation of DHA. Figure S4 Statistical analysis of field‐grown Camelina seed TAG data. Figure S5 Seed TAG profile from replicate analysis of pooled plot samples (100 mg of seed per replicate; n = 16 except for WT USA 2017 (n = 12), DHA1 USA 2017 (n = 11) and DHA1 Canada 2017 (n = 6) as determined by ESI‐MS/MS analysis (QTRAP 4000). Figure S6 Schematic representation of the Kennedy pathway and the biosynthetic routes to storage lipid (TAG). Table S1 Field trial location. Table S2 Field trial Rothamsted, USA & Canada weather condition. Table S3 Field trial Camelina growth season. Table S4 Statistical comparisons between DHA and WT. [file PBI-18-2280-s001.zip › pbi13385-sup-0001-Supinfo.pptx]

## Slide 1
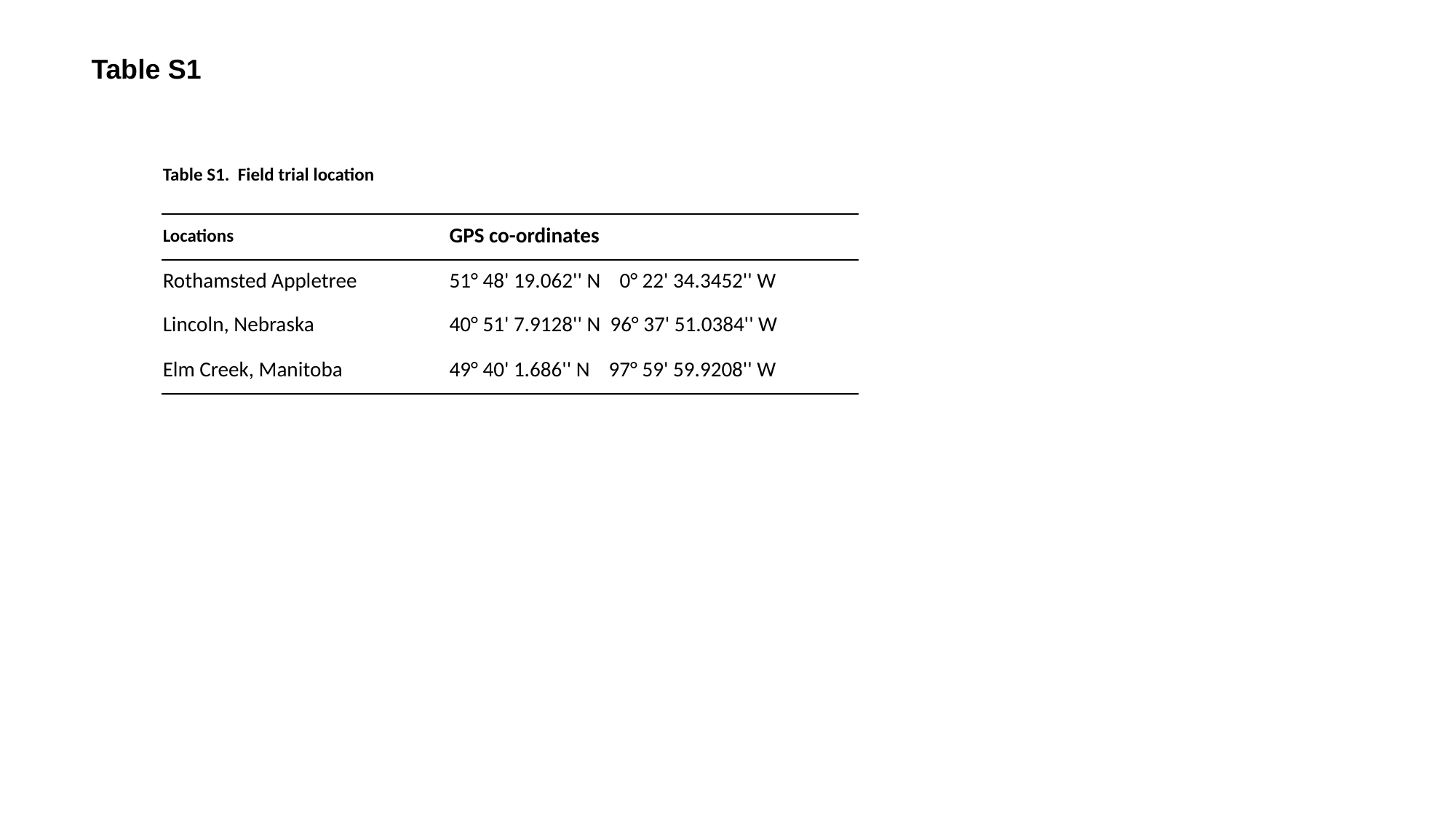

Table S1
| Table S1. Field trial location | |
| --- | --- |
| | |
| Locations | GPS co-ordinates |
| Rothamsted Appletree | 51° 48' 19.062'' N 0° 22' 34.3452'' W |
| Lincoln, Nebraska | 40° 51' 7.9128'' N 96° 37' 51.0384'' W |
| Elm Creek, Manitoba | 49° 40' 1.686'' N 97° 59' 59.9208'' W |

## Slide 2
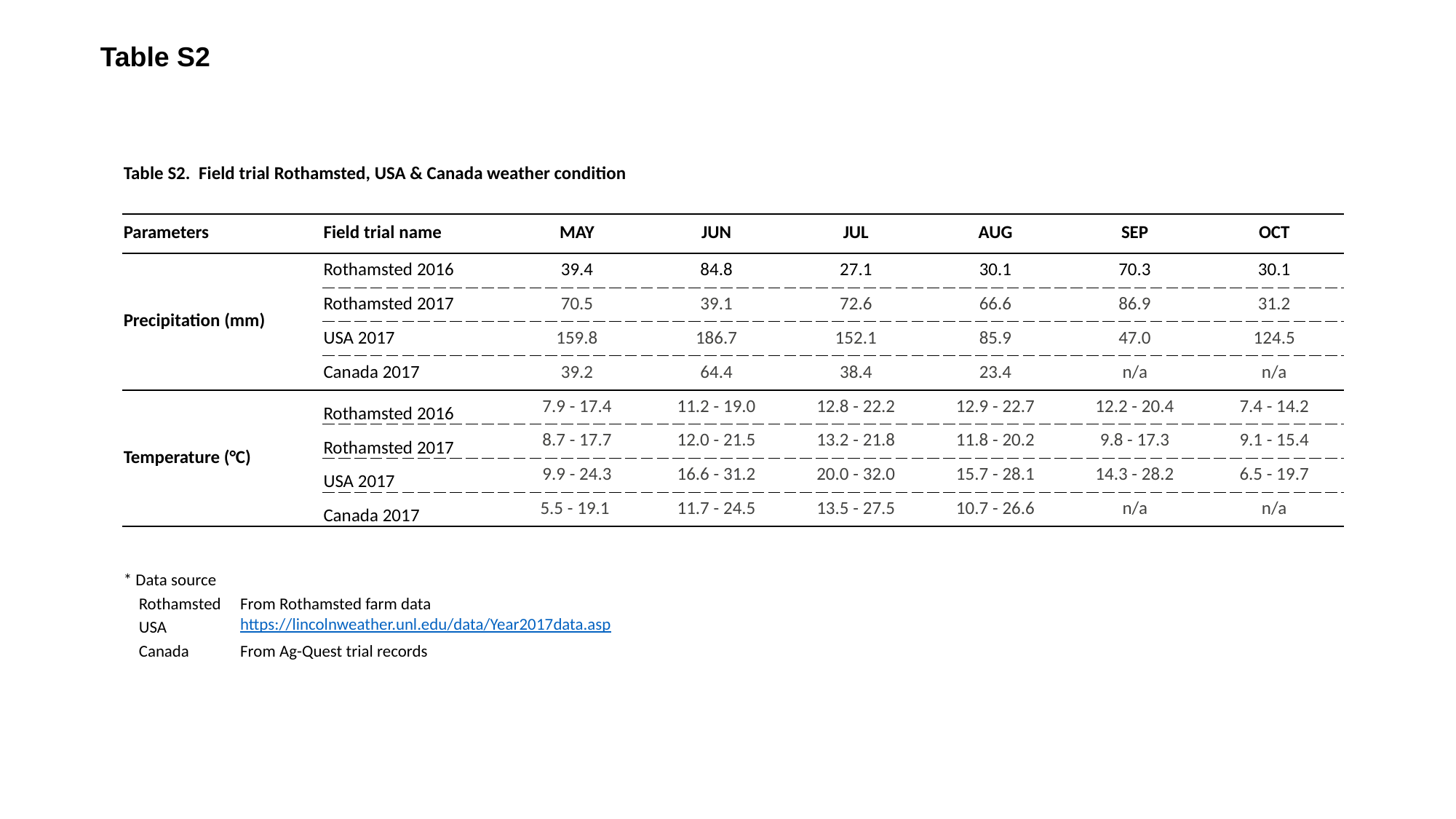

Table S2
| Table S2. Field trial Rothamsted, USA & Canada weather condition | | | | | | | |
| --- | --- | --- | --- | --- | --- | --- | --- |
| | | | | | | | |
| Parameters | Field trial name | MAY | JUN | JUL | AUG | SEP | OCT |
| Precipitation (mm) | Rothamsted 2016 | 39.4 | 84.8 | 27.1 | 30.1 | 70.3 | 30.1 |
| | Rothamsted 2017 | 70.5 | 39.1 | 72.6 | 66.6 | 86.9 | 31.2 |
| | USA 2017 | 159.8 | 186.7 | 152.1 | 85.9 | 47.0 | 124.5 |
| | Canada 2017 | 39.2 | 64.4 | 38.4 | 23.4 | n/a | n/a |
| Temperature (°C) | Rothamsted 2016 | 7.9 - 17.4 | 11.2 - 19.0 | 12.8 - 22.2 | 12.9 - 22.7 | 12.2 - 20.4 | 7.4 - 14.2 |
| | Rothamsted 2017 | 8.7 - 17.7 | 12.0 - 21.5 | 13.2 - 21.8 | 11.8 - 20.2 | 9.8 - 17.3 | 9.1 - 15.4 |
| | USA 2017 | 9.9 - 24.3 | 16.6 - 31.2 | 20.0 - 32.0 | 15.7 - 28.1 | 14.3 - 28.2 | 6.5 - 19.7 |
| | Canada 2017 | 5.5 - 19.1 | 11.7 - 24.5 | 13.5 - 27.5 | 10.7 - 26.6 | n/a | n/a |
| \* Data source | |
| --- | --- |
| Rothamsted | From Rothamsted farm data |
| USA | https://lincolnweather.unl.edu/data/Year2017data.asp |
| Canada | From Ag-Quest trial records |

## Slide 3
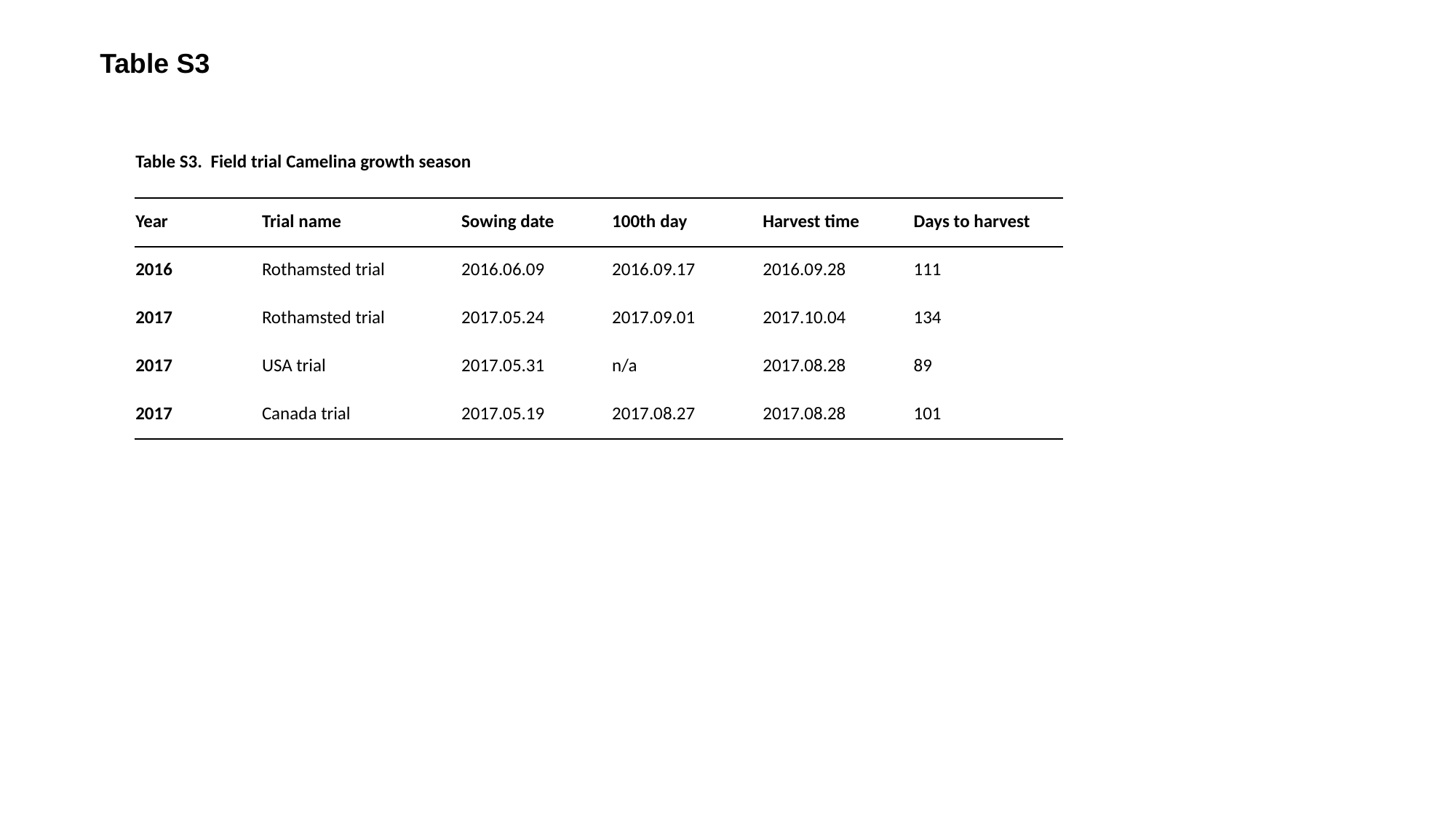

Table S3
| Table S3. Field trial Camelina growth season | | | | | |
| --- | --- | --- | --- | --- | --- |
| | | | | | |
| Year | Trial name | Sowing date | 100th day | Harvest time | Days to harvest |
| 2016 | Rothamsted trial | 2016.06.09 | 2016.09.17 | 2016.09.28 | 111 |
| 2017 | Rothamsted trial | 2017.05.24 | 2017.09.01 | 2017.10.04 | 134 |
| 2017 | USA trial | 2017.05.31 | n/a | 2017.08.28 | 89 |
| 2017 | Canada trial | 2017.05.19 | 2017.08.27 | 2017.08.28 | 101 |

## Slide 4
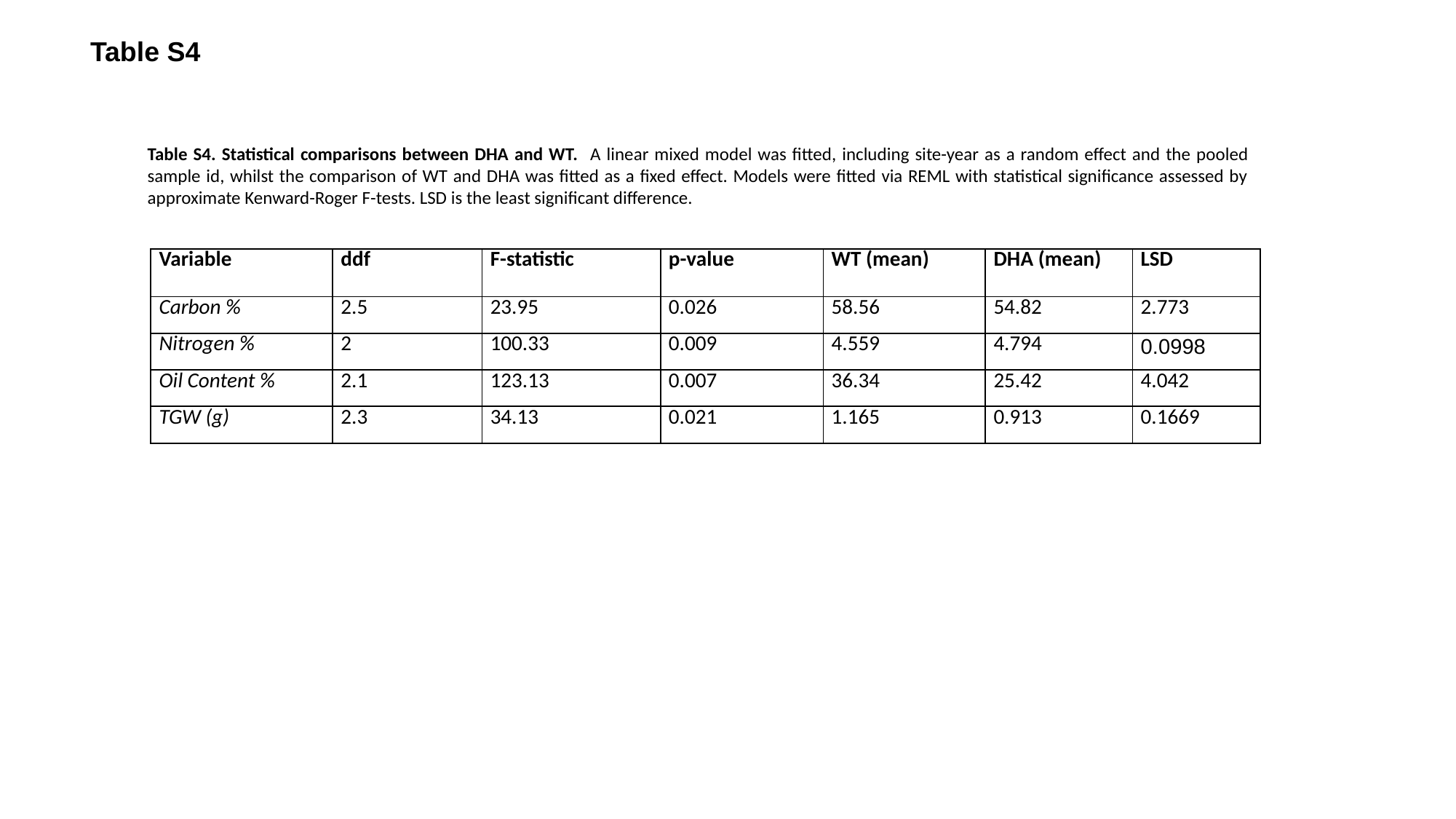

Table S4
Table S4. Statistical comparisons between DHA and WT. A linear mixed model was fitted, including site-year as a random effect and the pooled sample id, whilst the comparison of WT and DHA was fitted as a fixed effect. Models were fitted via REML with statistical significance assessed by approximate Kenward-Roger F-tests. LSD is the least significant difference.
| Variable | ddf | F-statistic | p-value | WT (mean) | DHA (mean) | LSD |
| --- | --- | --- | --- | --- | --- | --- |
| Carbon % | 2.5 | 23.95 | 0.026 | 58.56 | 54.82 | 2.773 |
| Nitrogen % | 2 | 100.33 | 0.009 | 4.559 | 4.794 | 0.0998 |
| Oil Content % | 2.1 | 123.13 | 0.007 | 36.34 | 25.42 | 4.042 |
| TGW (g) | 2.3 | 34.13 | 0.021 | 1.165 | 0.913 | 0.1669 |

## Slide 5
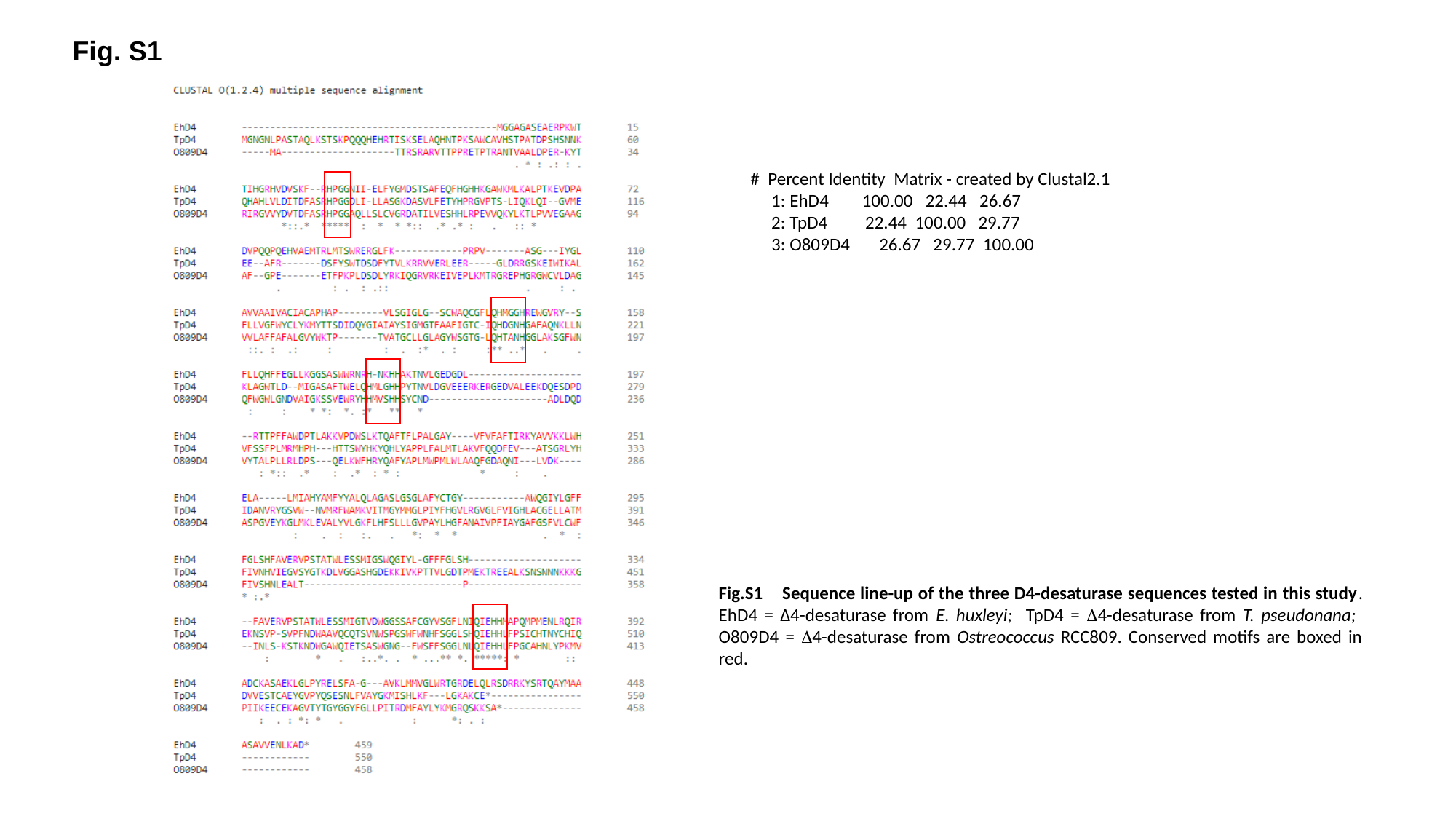

Fig. S1
# Percent Identity Matrix - created by Clustal2.1
 1: EhD4 100.00 22.44 26.67
 2: TpD4 22.44 100.00 29.77
 3: O809D4 26.67 29.77 100.00
Fig.S1 Sequence line-up of the three D4-desaturase sequences tested in this study. EhD4 = ∆4-desaturase from E. huxleyi; TpD4 = D4-desaturase from T. pseudonana; O809D4 = D4-desaturase from Ostreococcus RCC809. Conserved motifs are boxed in red.

## Slide 6
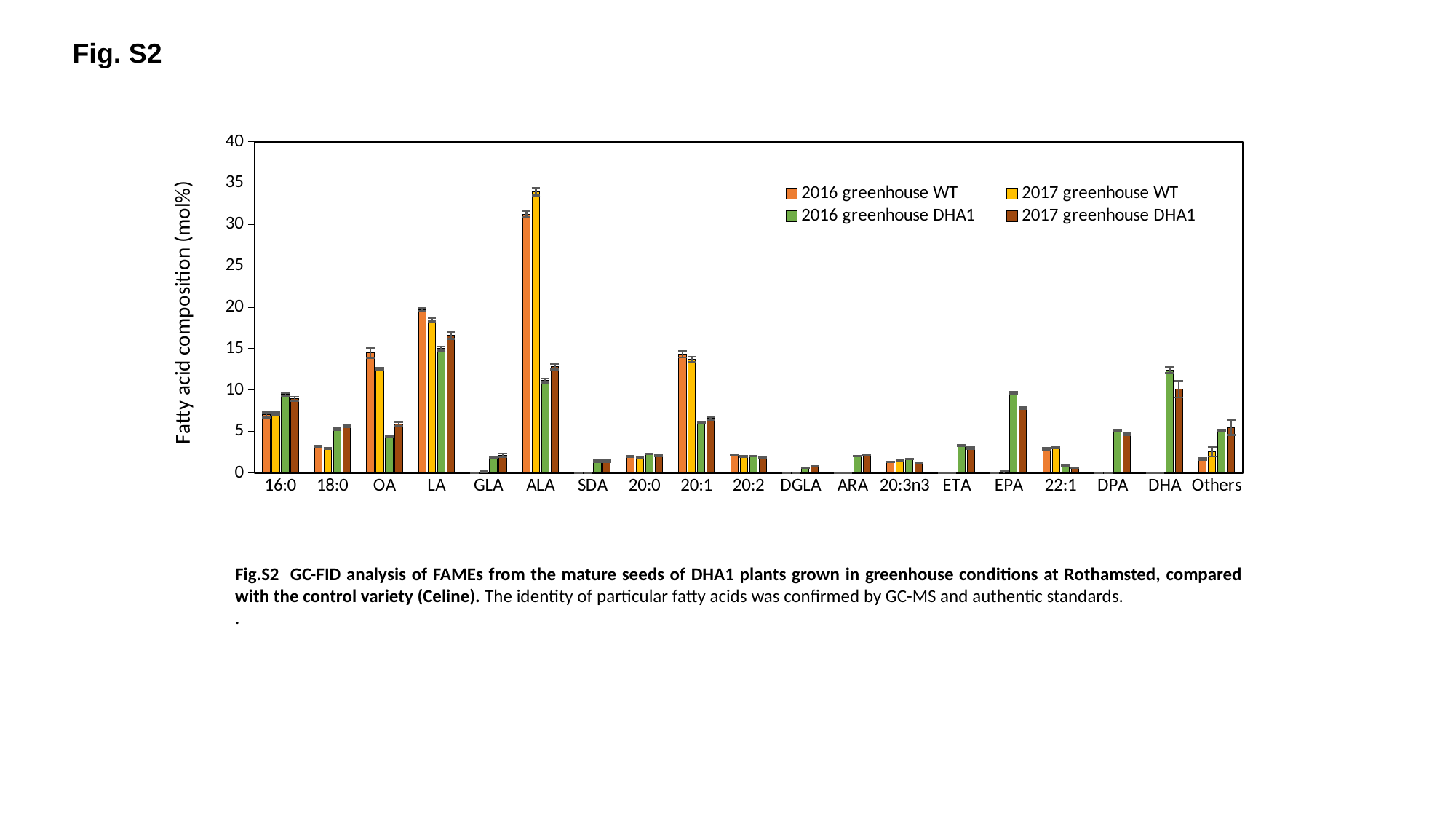

Fig. S2
### Chart
| Category | 2016 greenhouse WT | 2017 greenhouse WT | 2016 greenhouse DHA1 | 2017 greenhouse DHA1 |
|---|---|---|---|---|
| 16:0 | 7.013722264885651 | 7.178235662570355 | 9.49070129610419 | 8.940085393968094 |
| 18:0 | 3.2251580773915265 | 2.9475875428697105 | 5.2766909926474295 | 5.659301614011698 |
| OA | 14.520139338087128 | 12.53026555410826 | 4.4138507968329845 | 5.926455447842494 |
| LA | 19.704800874626606 | 18.51638604850278 | 15.023777091763488 | 16.622118407015805 |
| GLA | 0.0 | 0.16462249333700738 | 1.8502178816948711 | 2.110847747905965 |
| ALA | 31.255271338709587 | 33.98557720986469 | 11.170651664127838 | 12.83257854312178 |
| SDA | 0.0 | 0.0 | 1.4023164693758106 | 1.4150597368523214 |
| 20:0 | 1.9765116000124472 | 1.862861319096147 | 2.3024487449033013 | 2.073947344369188 |
| 20:1 | 14.345418433305053 | 13.715394572864163 | 6.148814154513579 | 6.584353067934559 |
| 20:2 | 2.0846966037402535 | 2.0044887492153203 | 2.0423262936990114 | 1.8996983865581183 |
| DGLA | 0.0 | 0.0 | 0.623864140929973 | 0.7980490731766311 |
| ARA | 0.0 | 0.0 | 2.060153313830271 | 2.1794539804619237 |
| 20:3n3 | 1.3136056611762028 | 1.4485794619562289 | 1.6800381974853273 | 1.1837540597259133 |
| ETA | 0.0 | 0.0 | 3.301058280730197 | 3.015455985397502 |
| EPA | 0.0 | 0.07964098679097295 | 9.671477237221513 | 7.839922682249487 |
| 22:1 | 2.9010647242806873 | 3.0248734609916603 | 0.8663789083093079 | 0.632090487302676 |
| DPA | 0.0 | 0.0 | 5.126063480481132 | 4.691764132283158 |
| DHA | 0.0 | 0.0 | 12.387271495368045 | 10.099649364275326 |
| Others | 1.6596110837848579 | 2.54148693783272 | 5.161899559981729 | 5.495414545547356 |Fig.S2 GC-FID analysis of FAMEs from the mature seeds of DHA1 plants grown in greenhouse conditions at Rothamsted, compared with the control variety (Celine). The identity of particular fatty acids was confirmed by GC-MS and authentic standards.
.

## Slide 7
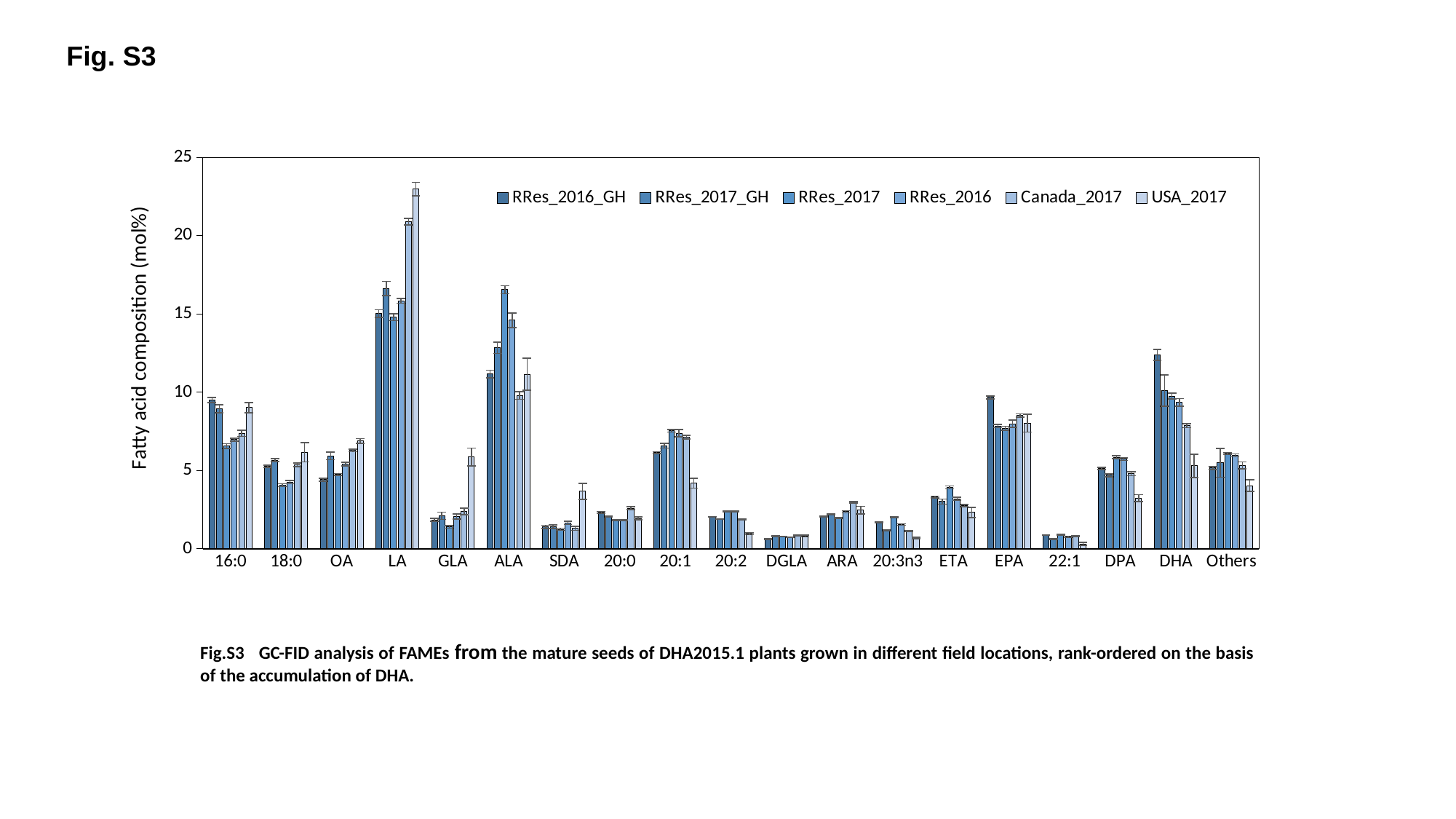

Fig. S3
### Chart
| Category | RRes_2016_GH | RRes_2017_GH | RRes_2017 | RRes_2016 | Canada_2017 | USA_2017 |
|---|---|---|---|---|---|---|
| 16:0 | 9.49070129610419 | 8.940085393968094 | 6.552461433893633 | 6.970424594926309 | 7.373412690613165 | 9.019880985701503 |
| 18:0 | 5.2766909926474295 | 5.659301614011698 | 4.062248706798763 | 4.2617950862926355 | 5.369993575342662 | 6.157693784270049 |
| OA | 4.4138507968329845 | 5.926455447842494 | 4.745097712374008 | 5.412162088581524 | 6.283435877855492 | 6.8866523643962525 |
| LA | 15.023777091763488 | 16.622118407015805 | 14.792536038662437 | 15.836638687009307 | 20.896673486039397 | 22.9751252577272 |
| GLA | 1.8502178816948711 | 2.110847747905965 | 1.397156862656161 | 2.0623596914212876 | 2.3826609665660787 | 5.867435705815072 |
| ALA | 11.170651664127838 | 12.83257854312178 | 16.551495458490326 | 14.59600992188613 | 9.780722596065356 | 11.149946257018662 |
| SDA | 1.4023164693758106 | 1.4150597368523214 | 1.2401844179534574 | 1.6578992355083841 | 1.3074857509772801 | 3.6662494535098435 |
| 20:0 | 2.3024487449033013 | 2.073947344369188 | 1.8169622357126471 | 1.8097150732251095 | 2.5908350217184357 | 1.9362003926718838 |
| 20:1 | 6.148814154513579 | 6.584353067934559 | 7.542230222699027 | 7.381971755715926 | 7.126347475470793 | 4.1859572923844235 |
| 20:2 | 2.0423262936990114 | 1.8996983865581183 | 2.3712635007243583 | 2.3801104637060844 | 1.8818205895180171 | 0.9579740797224034 |
| DGLA | 0.623864140929973 | 0.7980490731766311 | 0.7752602727128861 | 0.7386274315974125 | 0.8594152791921517 | 0.824753397937111 |
| ARA | 2.060153313830271 | 2.1794539804619237 | 1.95607715440729 | 2.3692446942898044 | 2.96814800362811 | 2.4734720877147924 |
| 20:3n3 | 1.6800381974853273 | 1.1837540597259133 | 2.024138974093944 | 1.5433949613732012 | 1.12254836860071 | 0.6812077676764458 |
| ETA | 3.301058280730197 | 3.015455985397502 | 3.9243166280455566 | 3.1970173196857012 | 2.7611188466552834 | 2.3161434195684367 |
| EPA | 9.671477237221513 | 7.839922682249487 | 7.6948014278559915 | 7.9843069712488255 | 8.51271819204225 | 8.02908461744485 |
| 22:1 | 0.8663789083093079 | 0.632090487302676 | 0.8853802052216644 | 0.7556066362975791 | 0.7897369652736489 | 0.3123982639807609 |
| DPA | 5.126063480481132 | 4.691764132283158 | 5.846313316300204 | 5.730486737011482 | 4.800646229736073 | 3.2341849079360423 |
| DHA | 12.387271495368045 | 10.099649364275326 | 9.752672838525356 | 9.34665818458698 | 7.859813661122089 | 5.296669929436534 |
| Others | 5.161899559981729 | 5.495414545547356 | 6.069402592872281 | 5.965570465636311 | 5.332466423583018 | 4.028970035087743 |Fig.S3 GC-FID analysis of FAMEs from the mature seeds of DHA2015.1 plants grown in different field locations, rank-ordered on the basis of the accumulation of DHA.

## Slide 8
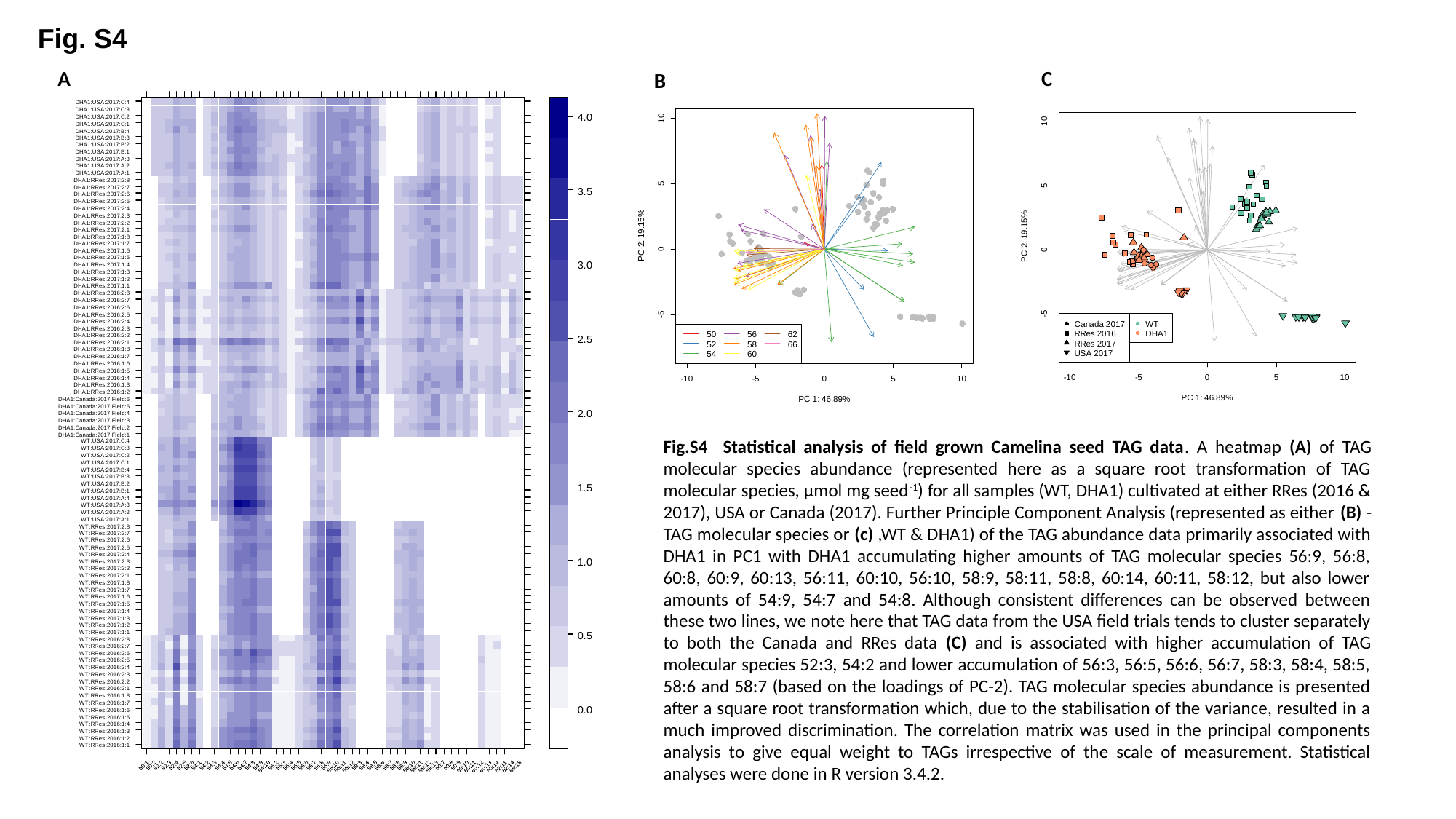

Fig. S4
C
A
B
Fig.S4 Statistical analysis of field grown Camelina seed TAG data. A heatmap (A) of TAG molecular species abundance (represented here as a square root transformation of TAG molecular species, µmol mg seed-1) for all samples (WT, DHA1) cultivated at either RRes (2016 & 2017), USA or Canada (2017). Further Principle Component Analysis (represented as either (B) -TAG molecular species or (c) ,WT & DHA1) of the TAG abundance data primarily associated with DHA1 in PC1 with DHA1 accumulating higher amounts of TAG molecular species 56:9, 56:8, 60:8, 60:9, 60:13, 56:11, 60:10, 56:10, 58:9, 58:11, 58:8, 60:14, 60:11, 58:12, but also lower amounts of 54:9, 54:7 and 54:8. Although consistent differences can be observed between these two lines, we note here that TAG data from the USA field trials tends to cluster separately to both the Canada and RRes data (C) and is associated with higher accumulation of TAG molecular species 52:3, 54:2 and lower accumulation of 56:3, 56:5, 56:6, 56:7, 58:3, 58:4, 58:5, 58:6 and 58:7 (based on the loadings of PC-2). TAG molecular species abundance is presented after a square root transformation which, due to the stabilisation of the variance, resulted in a much improved discrimination. The correlation matrix was used in the principal components analysis to give equal weight to TAGs irrespective of the scale of measurement. Statistical analyses were done in R version 3.4.2.

## Slide 9
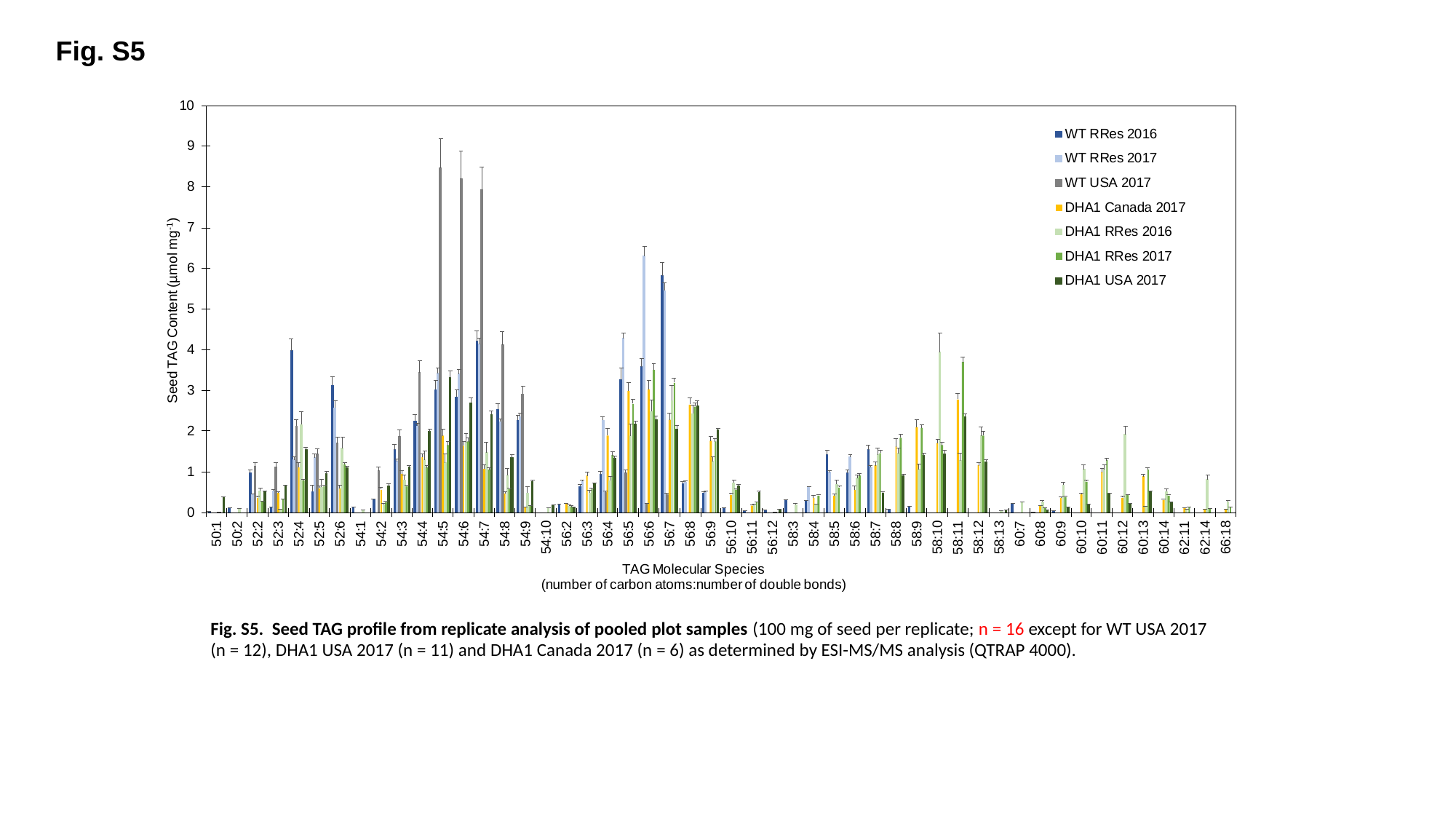

Fig. S5
Fig. S5. Seed TAG profile from replicate analysis of pooled plot samples (100 mg of seed per replicate; n = 16 except for WT USA 2017 (n = 12), DHA1 USA 2017 (n = 11) and DHA1 Canada 2017 (n = 6) as determined by ESI-MS/MS analysis (QTRAP 4000).

## Slide 10
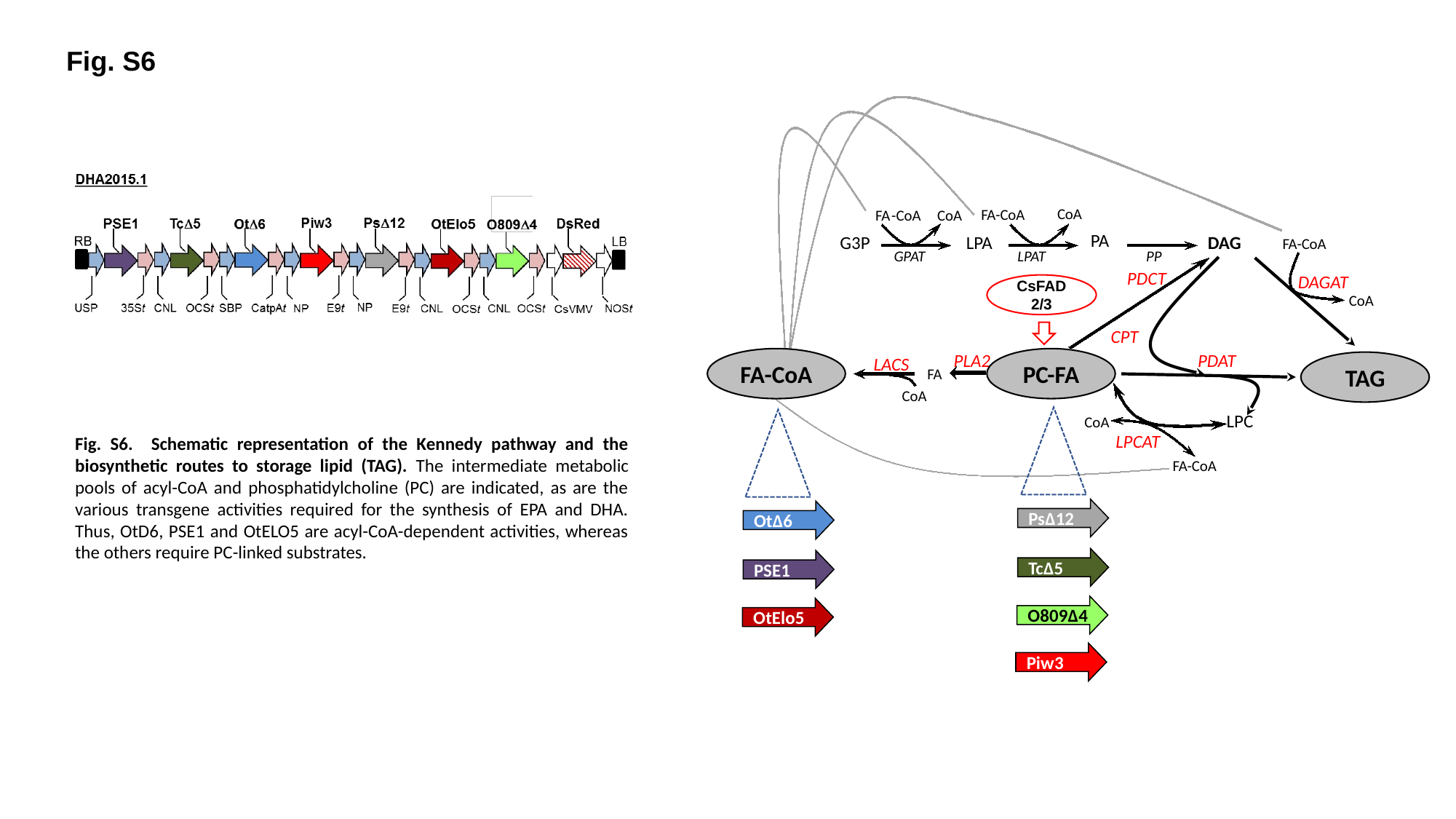

Fig. S6
CoA
FA-CoA
FA
-
CoA
CoA
PA
G3P
LPA
DAG
FA-CoA
GPAT
LPAT
PP
PDCT
DAGAT
CoA
CPT
PC-FA
FA-CoA
PLA2
PDAT
TAG
LACS
 FA
CoA
LPC
CoA
LPCAT
FA-CoA
PsΔ12
TcΔ5
O809Δ4
Piw3
OtΔ6
PSE1
OtElo5
CsFAD2/3
Fig. S6. Schematic representation of the Kennedy pathway and the biosynthetic routes to storage lipid (TAG). The intermediate metabolic pools of acyl-CoA and phosphatidylcholine (PC) are indicated, as are the various transgene activities required for the synthesis of EPA and DHA. Thus, OtD6, PSE1 and OtELO5 are acyl-CoA-dependent activities, whereas the others require PC-linked substrates.
